# Ontology-aware deep learning enables ultrafast and interpretable source tracking among sub-million microbial community samples from hundreds of niches

**DOI:** 10.1186/s13073-022-01047-5

**Published:** 2022-04-26

**Authors:** Yuguo Zha, Hui Chong, Hao Qiu, Kai Kang, Yuzheng Dun, Zhixue Chen, Xuefeng Cui, Kang Ning

**Affiliations:** 1 Key Laboratory of Molecular Biophysics of the Ministry of Education, Hubei Key Laboratory of Bioinformatics and Molecular-imaging, Center of AI Biology, Department of Bioinformatics and Systems Biology, College of Life Science and Technology, Huazhong University of Science and Technology, Wuhan, 430074 Hubei China; 2 School of Mathematics and Statistics, Huazhong University of Science and Technology, Wuhan, 430074 Hubei China; 3 Institute for Interdisciplinary Information Sciences, Tsinghua University, Beijing, 100084 China; 4 School of Computer Science and Technology, Shandong University, Qingdao, 266237 Shandong China

**Keywords:** Ontology-aware Neural Network (ONN), Microbial source tracking (MST), Deep learning, Ultrafast, Biomes

## Abstract

The taxonomic structure of microbial community sample is highly habitat-specific, making source tracking possible, allowing identification of the niches where samples originate. However, current methods face challenges when source tracking is scaled up. Here, we introduce a deep learning method based on the Ontology-aware Neural Network approach, ONN4MST, for large-scale source tracking. ONN4MST outperformed other methods with near-optimal accuracy when source tracking among 125,823 samples from 114 niches. ONN4MST also has a broad spectrum of applications. Overall, this study represents the first model-based method for source tracking among sub-million microbial community samples from hundreds of niches, with superior speed, accuracy, and interpretability. ONN4MST is available at https://github.com/HUST-NingKang-Lab/ONN4MST.

**Supplementary Information:**

The online version contains supplementary material available at 10.1186/s13073-022-01047-5.

## Background

With the rapid accumulation of microbial community samples from various niches (biomes) around the world, as well as the huge volume of sequencing data deposited into public databases, such as those from the “Human Microbiome Project” [1, 2] and the “Earth Microbiome Project” [3, 4], knowledge about microbial communities and their influence on the environment and human health has grown rapidly [5, 6]. Such massive microbial community samples provide the opportunity to study the inconspicuous evolution and ecological patterns among microbial communities, especially habitat-specific patterns.

Taxonomic composition of a microbial community sample is usually represented by hierarchically-structured taxa and their relative abundances (also referred to as the community structure), and these taxa are functioning in concert to maintain the stability of the microbial community and its adaptation to the specific environment (also referred to as the niche or biome) [7, 8]. Biomes are well organized into a hierarchical structure with multiple layers [9], and the hierarchy is reflected by the parent-child relationships between biomes (i.e., “Human-Digestive system”). In MGnify project’s definition (for which we follow), layer one is the highest layer containing only one biome “Root,” and layer six is the lowest (bottom) layer containing biomes such as “Fecal.” The hierarchical structure is also considered as an ontology in our work, which is widely accepted by current microbiome researches, and profiled in both MGnify [8] and GOLD [9]. In general, microbial community samples from the same biome tend to have similar community structures, while such similarities are highly dependent on the biome layers.

The rapid accumulation of microbial community samples has provided the opportunity to investigate the interactions among microbes, human health, and environment, while they have created an enormous hurdle for characterizing the potential inputs from other associated biomes, calling for fast and accurate source tracking [10–12]. Considerable attention has been paid to explore the interactions on small scales, such as the disease diagnosis, early development, pregnancy, and immigration, while integrative, large-scale, and scalable investigations have been understudied. Such investigation is challenging for reasons: firstly, as the number of samples easily exceeds millions [8], while the number of niches exceeds hundreds, the microbial source tracking has already become a very complex task. Secondly, the noises that existed in the rich-sourced data might hire important patterns invisible for traditional methods. Coupled with the fact that many biomes are dependent with each other, previous models would be theoretically inapplicable.

Several methods for microbial community source tracking have already been proposed [12–16]. They can generally be divided into two categories: distance-based methods such as Jensen-Shannon Divergence (JSD) [17], Striped UniFrac [14], and Meta-Prism [18]; unsupervised machine learning methods such as SourceTracker [16, 19] based on Bayesian algorithm and FEAST [12] based on Expected-Maximization algorithm. However, the limitations of these methods are apparent: Firstly, currents methods are suitable in small-scale source tracking studies, yet unsupervised methods face a tradeoff between source tracking accuracy and efficiency [12], thus are limited to source track for only a few hundreds of samples from a handful of biomes within a reasonable time. Secondly, when the background of source tracking research occurs in an extremely complex environment, researchers usually have little background knowledge about samples, leading to low source tracking accuracy. Under all of these situations, the knowledge about actual source biomes is often hidden in the large fraction of unknowns.

To address these limitations, we developed ONN4MST, an Ontology-aware Neural Network (ONN) computational model for microbial source tracking. The ONN model employs a novel ontology-aware approach that encourages prediction satisfying the “biome ontology.” In other words, the ONN model can utilize the biome ontology information to model the dependencies between biomes, and estimate the proportion of various biomes in a community sample. Published studies about ontology-aware hierarchical classifiers have shown advantages of encoding ontology structure into a neural network, such as PHENOstruct [20] and DeepPheno [21]. It is worth noting that ONN4MST uses a large amount of data (125,823 samples from 114 biomes, accounting for more than half of the MGnify project, as of the year 2020) to train the model, which allows it to be applicable for source tracking samples from many biomes. ONN4MST has provided an ultrafast (less than 0.1 s) and accurate (AUC higher than 0.97 in most cases) solution for searching a sample against a dataset containing hundreds of potential biomes and millions of samples, and also out-performed state-of-the-art methods in scalability and stability. The ability of ONN4MST on knowledge discovery is also demonstrated in various source tracking applications: it enables source tracking of samples whose niches are previously less studied or unknown, detection of microbial contaminants, as well as identification of similar samples from ontologically-remote biomes, showing the unique importance of ONN4MST in knowledge discovery from a huge amount of microbial community samples of heterogeneous biomes.

## Methods

### Datasets

We evaluated the performances of ONN4MST and other source tracking methods based on five different datasets (Additional file 1: Table S1). These five datasets comprise samples from different niches, which are representative of high-quality samples in public resources.

The “Combined dataset” consists of 125,823 microbial community samples collected from the EBI MGnify database [8] (https://www.ebi.ac.uk/metagenomics/), accessed as of January 2020 (Additional file 1: Table S1). This is a comprehensive dataset containing samples from 114 biomes (Additional file 1: Table S2), and the 125,823 microbial community samples represent more than half of the samples in EBI MGnify (as of January 1, 2020). These samples contain taxonomic information for 225 phyla, 6232 families, 16,081 genera, and 45,477 species.

The “Human dataset” consists of 53,553 microbial community samples selected from the Combined dataset, representing a subset of samples from the human niches (Additional file 1: Table S1). Specifically, these samples are collected under these biomes: “Root-Host_associated-Human-Skin,” “Root-Host_associated-Human-Circulatory_system,” “Root-Host_associated-Human-Digestive_system,” and “Root-Host_associated-Human-Reproductive_system” (biomes at a higher layer). This dataset contains 53,553 samples from a total of 25 biomes. These samples contain taxonomic information for 204 phyla, 2801 families, 6523 genera, and 16,135 species.

The “Water dataset” consists of 27,667 microbial community samples selected from the Combined dataset, representing a subset of samples from the water niches (Additional file 1: Table S1). Specifically, these samples are collected under these biomes: “Root-Environmental-Aquatic-Freshwater,” “Root-Environmental-Aquatic-Marine,” and “Root-Environmental-Aquatic-Non-marine_Saline_and_Alkaline” (biomes at a higher layer). This dataset contains 27,667 samples from a total of 44 biomes. These samples contain taxonomic information for 222 phyla, 6040 families, 15,261 genera, and 36,406 species.

The “Soil dataset” consists of 11,528 microbial community samples selected from the Combined dataset, representing a subset of samples from the soil niches (Additional file 1: Table S1). Specifically, these samples are collected under these biomes: “Root-Environmental-Terrestrial-Soil,” and “Root-Host_associated-Plants-Rhizosphere” (biomes at a higher layer). This dataset contains 11,528 samples from a total of 16 biomes. These samples contain taxonomic information for 201 phyla, 2962 families, 6753 genera, and 12,769 species.

These three datasets (Human, Water, and Soil datasets) were designed with several reasons in consideration. Firstly, these three datasets are representative enough and frequently used subsets from the Combined dataset. Secondly, these three datasets are also distinct, since the Alpha diversity of samples from each of these datasets is significantly different from the other two: while samples from soil niches are considered more complicated, those from human and water niches are considered less so. Finally, samples from these niches are more comprehensively explored than other less studied niches, and they are of relatively higher quality of samples from these three niches.

The “FEAST dataset” consists of 10,270 microbial community samples selected from the datasets used in the Lax et al. [12] (Additional file 1: Table S1). Specifically, these samples are all collected from three biomes (“Root-Host_associated-Human,” “Root-Host_associated-Human-Digestive_system-Large_intestine-Fecal” and “Root-Mixed”). These samples contain taxonomic information for 133 phyla, 1118 families, 3389 genera, and 5762 species. The “FEAST dataset” is the smallest dataset used in this study, and it is the simplest dataset with regard to the number of biomes involved. Yet it is a dataset of unique importance, as the source tracking methods evaluated in this study could be benchmarked on this medium-sized and credible human gut dataset [12, 16] for a fair assessment of accuracy and efficiency.

The dataset used in the case study of centenarian was collected and studied by Bian et al. [22] (accession number SRP107602) and Biagi et al. [23] (from multiple sources). The dataset used in the case study of exploring the association of niche and microbes was from the EBI MGnify database [8] (Study MGYS00001056). The dataset used in the case study of detecting microbial contamination in a built environment was collected and studied by Lax et al. [24] (accession number ERP005806). The dataset used in the case study of less studied biomes was collected and studied by Alsalah et al. [25] (accession number PRJEB9501). The dataset used in the case study of bird biome was from EBI MGnify database [8] (Study MGYS00005593). The dataset used in the case study of Hadza people’s gut microbial communities was collected and studied by Samuel et al. [26] (accession number PRJNA392012, PRJNA392180).

### Biome ontology

We constructed a comprehensive biome ontology using 114 biomes (Additional file 1: Table S2) collected from the EBI MGnify database [8] (https://www.ebi.ac.uk/metagenomics/biomes). In this process, we organized the biome ontology as a tree, by treating a biome with multiple parent biomes in the higher layer (e.g., “Human-Digestive_system” and “Mammal-Digestive_system”) as separate biomes. Next, the ontology tree containing 6 layers and 133 nodes (representing 114 biomes) was constructed, by using Python-3.7.4 and Treelib-1.5.5. As a result, each biome was represented by at least one node in the ontology tree. The ontology tree has “Root” at the first layer, biomes (nodes) including “Environmental,” “Host_associated,” and “Engineered” at the second layer, and 7, 22, and 56 biomes (nodes) at the third to fifth layers respectively, with 43 biomes (nodes) including “Coral reef,” “Fecal,” and “Saliva” at the bottom (sixth) layer (Additional file 1: Table S2).

### Sample labeling

In all experiments, we used microbial samples each with a label annotated by using 6-layers biome ontology to validate our model. For example, there are 22 samples labeled as “Root-Host_associated-Human-Digestive_system-Oral-Throat” in the Combined dataset (by separating different layers with the “−” symbol).

### Data representation

**Fig. 1:**
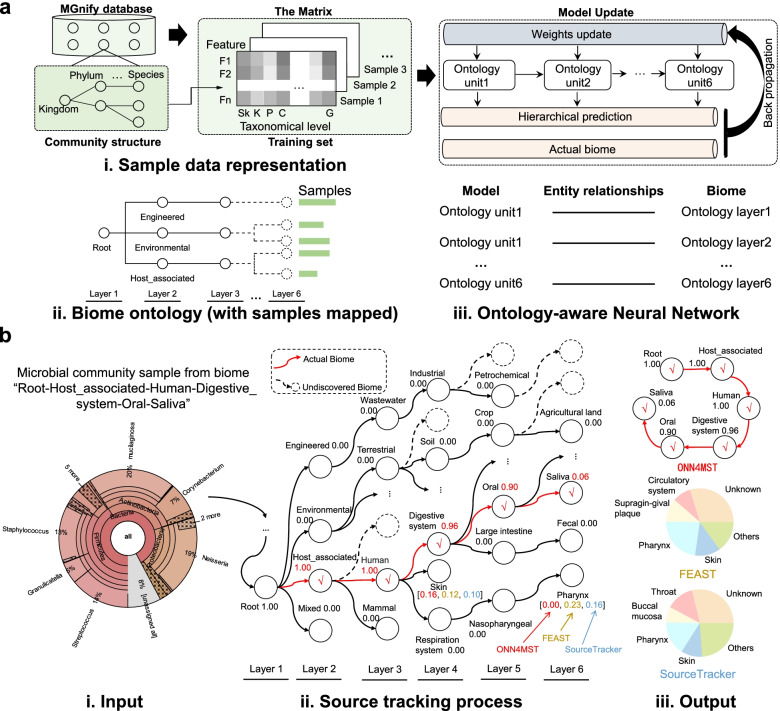
Building and using the ONN model for microbial source tracking. **a** The sample data representation and training process of ONN model. (i) Sample data are transformed into the Matrix. With the Matrix, each column represents a taxonomic level and each row represents a feature; (ii) In parallel, samples are mapped to biome ontology according to their niches; (iii) The model is built and updated according to both samples’ abundance matrices and biome ontology information. More details about building, testing, and using the ONN model for source tracking are illustrated in Supplementary Figs. 1 and 2. **b** An illustrated example of microbial source tracking procedure using ONN4MST. (i) The input is the community structure of a real microbial community sample (this sample is from the biome “Root-Host_associated-Human-Digestive_system-Oral-Saliva”) that has been preprocessed and the Matrix has been provided into the model; (ii) Source tracking process at different layers. The red arrows indicate the search process from layer 1 to layer 6, accompanied with source contribution annotated in red. To compare with the procedure of ONN4MST, the yellow and blue arrows indicated the source tracking results (among the overall top 5 sources) of FEAST and SourceTracker, together with their source contributions, respectively. The actual biome is annotated by a red checkmark; (iii) The predicted biomes (with source contributions) by ONN4MST, FEAST, and SourceTracker

ONN4MST takes species abundance table as input, which can be generated by using standard programs (e.g., Qiime) based on 16S rRNA data or metagenomics data. Then, we generated the Matrix for each microbial community sample based on the species abundance table, so that the abundances for all taxa at seven taxonomic levels including super-kingdom, kingdom, phylum, class, order, family, and genus (simply referred to as “sk,” “k,” “p,” “c,” “o,” “f,” and “g”) can be retained. The abundance of taxa at different levels was filled in the Matrix (Fig. 1). Within the Matrix, seven columns respectively represent seven taxonomic levels. And 44,668 rows respectively represent relative abundance for 44,668 taxa (also referred to as features). For a detailed description and an example of the data representation, see Additional files 2 and 3.

### Feature selection

To improve the efficiency and accuracy of ONN4MST, we conducted feature selection by using a random forest regression model (Python-3.7.4 and Scikit-learn-0.22.1). An abundance-based pre-filtering and an importance-based selection were performed in sequential order. In doing so, we treated each row (representing the abundances of a taxon, see Additional file 2) of the Matrix as a feature. Then, a series of adaptive thresholds ($C\overline{R_l}$ and $C\overline{I_l}$) were applied to different taxon levels, in which $\overline{R_l}$ and $\overline{I_l}$ stand respectively for the relative abundance and the feature importance. level ∈ {*sk*, *k*, *p*, *c*, *o*, *f*, *g*} and the coefficient *C* was set to 0.001. As a result, we have selected 1,462 features with relative abundance and feature importance above the thresholds from all 44,668 features involved in this study.

### The ONN4MST model

The training and query processes are built based on two data structures: microbiome samples represented by its community structure, as well as the biome ontology represented by a hierarchy *O*. In the training process, we employed an Ontology-aware Neural Network to learn a mapping *M*(*s*) from a series of source samples *s* ∈ *D* to their biome sources ${x}_s=\left({x}_s^2,\dots, {x}_s^6\right)$, with source contributions ${y}_s=\left({y}_s^2,\dots, \kern0.5em {y}_s^6\ \right)$, where *D* is the source sample dataset, ${x}_s^i$ is source biome, ${y}_s^i$ is source contribution for source sample *s* in the *i*th layer of *O*. In the query process, we apply *M* on query *q* to determine the source biomes and maximum contributions for the query sample *q*: Considering a query sample *q*, we quantify contributions *y*_*q*_(*x*) from every biome source *x* to *q*, and determine the biome source ${x}_q=\left({x}_q^2,\dots {x}_q^6\right)$ that could maximize ${y}_q=\left({y}_q^2,\dots, \kern0.5em {y}_q^6\ \right)$ as the source tracking result.

### Architecture of the Ontology-aware Neural Network

The architecture of the ONN could be described in four functional layers, including feature extracting layer, feature encoding layer, feature incorporating layer, and ontology-aware layer (Additional file 1: Fig. S1). The feature extracting layer (input layer) is used for extracting the basic feature of microbial community samples. The feature extracting layer is a fully-connected layer with ReLU activation. It accepts microbial community samples represented by the Matrix, extracts the feature information from the Matrix, and delivers them to the feature encoding layer. The feature encoding layer is used for encoding ontology-layer-specific features of microbial community samples. The feature encoding layer is a fully-connected layer with ReLU activation. It accepts the output of the feature extraction layer and encodes ontology-layer-specific feature information for each of the six biome ontology layers. The feature incorporating layer is used for incorporating inter-layer information. The feature incorporating layer is a fully-connected layer with ReLU activation, which serves for inter-layer information incorporation. The ontology-aware layer (output layer) is used for ontology walk-through and source contribution calculation. The ontology-aware layer is a fully-connected layer with Sigmoid activation. It accepts the output of the feature incorporating layer and computes the contribution of all biome sources on its corresponding biome ontology layer.

### Training and testing

We used Tensorflow-1.14 [27] to build and train the ONN model. The model was trained on a computational platform comprising Intel(R) Xeon(R) CPU E7-4809 v3 @ 2.00GHz CPU (64 cores in total) with 315 GB RAM and Nvidia Tesla K80 GPU with 12 GB RAM. We chose 8-fold cross-validation for model training and testing. For each dataset, we randomly split it into 8 folds, each fold including a training set (87.5%) and a testing set (12.5%). For each fold, the model was trained (in batches of 512 samples) for 30,000 iterations or until training accuracy converged, and the model with the highest accuracy on the training set was selected for testing. The results on the testing set are organized in the form of a hierarchical prediction (with prediction results from 2nd to 6th layers), which would then be evaluated.

### Other methods used in this study

Three distance-based methods, JSD [17], Striped UniFrac [14], and Meta-Prism [18]; two unsupervised machine learning methods, Expected-Maximization-based method FEAST [12] and Bayesian-based method SourceTracker [16]; and our supervised deep learning method (ONN4MST) were applied for microbial source tracking. In this study, the source tracking results (predicted biomes) of multiple methods were compared against the microbial community samples’ actual source (actual biomes).

The distance-based methods are based on a pair-wise calculation of sample distances, and such methods depend heavily on the presence of species and their relative abundance for individual samples, regardless of weighted or unweighted scoring functions used. Among distance-based methods, JSD does not consider the phylogenetic relationships among species, while methods such as Striped UniFrac and Meta-Prism do (we have used Meta-Prism 2.0 for comparison in this study). However, distance-based methods have a binomial increase in time cost with the increase in the number of samples.

Unsupervised methods for microbial community sample comparison are based on profile-based statistical models, either the Bayesian model used in the SourceTracker method or the Expected-Maximization (EM) model used in the FEAST method. Unsupervised methods are typically more accurate than distance-based methods. However, since unsupervised methods still do not consider the intricate but important patterns of a set of samples from similar niches, their tolerance to noisy signals in samples is not high, hence potentially would lead to biased mismatches. Details about the source tracking methods other than ONN4MST used in this study are provided in Additional file 3.

### Hierarchical prediction

In order to carry out a comparison of ONN4MST against other methods at different layers of biome ontology, all other methods were remolded, so that the prediction results of these methods (excluding ONN4MST) at different layers could be produced. Based on the source contributions of biomes at the sixth (bottom) layer, the source contributions of biomes for other layers were computed using ${P}_f={\sum}_{f_c\in {C}_f}{P}_{f_c}$. Where *P*_*f*_ is a source contribution for *f*, *C*_*f*_ is a set of children biomes for biome source *f* in the biome ontology. *f*_*c*_ is a child biome of *f*. We used NumPy-1.18.1 and Treelib-1.5.5 in the process.

### Benchmarking measures

To benchmark and compare the results based on ONN4MST and the other five methods, we used these measures:

1 $${\mathit{TP}}_f\left(t\right)={\textstyle\sum_i}I\left(f\in P_i\left(t\right)\wedge f\in T_i\right)$$

2 $${\mathit{TN}}_f\left(t\right)={\textstyle\sum_i}I\left(f\not\in P_i\left(t\right)\wedge f\not\in T_i\right)$$

3 $${\mathit{FP}}_f\left(t\right)={\textstyle\sum_i}I\left(f\in P_i\left(t\right)\wedge f\not\in T_i\right)$$

4 $${\mathit{FN}}_f\left(t\right)={\textstyle\sum_i}I\left(f\not\in P_i\left(t\right)\wedge f\in T_i\right)$$

5 $${ TP R}_f(t)=\frac{TP_f(t)}{TP_f(t)+{FN}_f(t)}$$

6 $${ FP R}_f(t)=\frac{FP_f(t)}{FP_f(t)+{TN}_f(t)}$$

7 $$\mathit{TPR}\left(t\right)=\frac1F{\textstyle\sum_{f=1}^F}{\mathit{TPR}}_f\left(t\right)$$

8 $$\mathit{FPR}\left(t\right)=\frac1F{\textstyle\sum_{f=1}^F}{\mathit{FPR}}_f\left(t\right)$$

where *f* is a biome source, *P*_*i*_(*t*) is a set of predicted biomes for a microbial community sample *i* and threshold *t* ∈ [0, 1] with a step size of 0.01, *T*_*i*_ is a set of actual biomes for a sample *i*, *F* is the total number of biomes, and *I* is a logical operation function, the value of *I* is 1 when the result of the logical operation is TRUE, else 0.

Four evaluation metrics (*Accuracy*, *Precision*, *Recall*, and *F*_max_) were introduced. These evaluation metrics are computed with the following formulas:

9 $$Accuracy(t)=\kern0.5em \frac{TP_f(t)+{TN}_f(t)}{TP_f(t)+{FP}_f(t)+{TN}_f(t)+{FN}_f(t)}$$

10 $${Precision}_f(t)=\frac{TP_f(t)}{TP_f(t)+{FP}_f(t)}$$

11 $${Recall}_f(t)=\kern0.5em \frac{TP_f(t)}{TP_f(t)+{FN}_f(t)}$$

where *TP* is true positive, *TN* is true negative, *FP* is false positive, and *FN* is false negative. Subsequently, we compute *F*1 for threshold *t* ∈ [0, 1] with a step size of 0.01 by using the average precision and average recall for all actual biomes that we predicted at least one time. Then, we select the maximum *F*1 as *F*_max_. These evaluation metrics are computed with the following formulas:

12 $$\textit{AvgPrecision}\left(t\right)=\frac1F{\textstyle\sum_{f=1}^F}{\textit{Precision}}_f\left(t\right)$$

13 $$\textit{AvgRecall}\left(t\right)=\frac1F{\textstyle\sum_{f=1}^F}{\textit{Recall}}_f\left(t\right)$$

14 $${F}_{max}=\underset{t}{\mathit{\max}}\left\{\frac{2\bullet AvgPrecision(t)\bullet AvgRecall(t)}{AvgPrecision(t)+ AvgRecall(t)}\right\}$$

Then, ROC (Receiver Operating Characteristic) curves, which are based on contrasting the true positive rate (TPR) against the false positive rate (FPR), were plotted. AUC (Area Under the Curve) reflects the ability of model to correctly predict the biomes (sources) of microbial community samples. AUC is calculated with the following formula:

15 $$AUC={\int}_0^1 TPR(t)\left(-{FPR}^{\prime }(t)\right) dt$$

## Results

### Ontology-aware Neural Network

ONN4MST uses an Ontology-aware Neural Network (ONN) model for source tracking. When training the model, all training samples’ community structures are decoded, each converted to a matrix containing the taxa at different taxonomic levels and their relative abundances (simply referred to as the Matrix). The ONN model uses the Matrix as input and reshapes it into tensors which point to biomes at every different layer of the biome ontology. To fit the structure of biome ontology, the ONN model uses multiple ontology units, each belonging to one of the six specific layers of biome ontology (Fig. 1a). The architecture, the training procedure, and the evaluation procedure of the ONN model are illustrated in Additional file 1: Fig. S1 and described in the “Methods” section.

The source tracking procedure of ONN4MST is illustrated in Fig. 1b. Since ONN4MST is the first method available that could source track the samples at different layers of biome ontology, the search scheme of ONN4MST is completely different from other methods (Fig. 1b). While ONN4MST goes through the biome ontology to find the best possible source along different layers, other methods such as FEAST and SourceTracker treat all biomes as anarchically equal. The overall scheme of building the ONN model and using ONN4MST for source tracking is illustrated in Additional file 1: Fig. S2. Note that the contributions of every known biome would be estimated by the ONN model, respectively.

### General model enables accurate source tracking with high scalability and stability

**Fig. 2:**
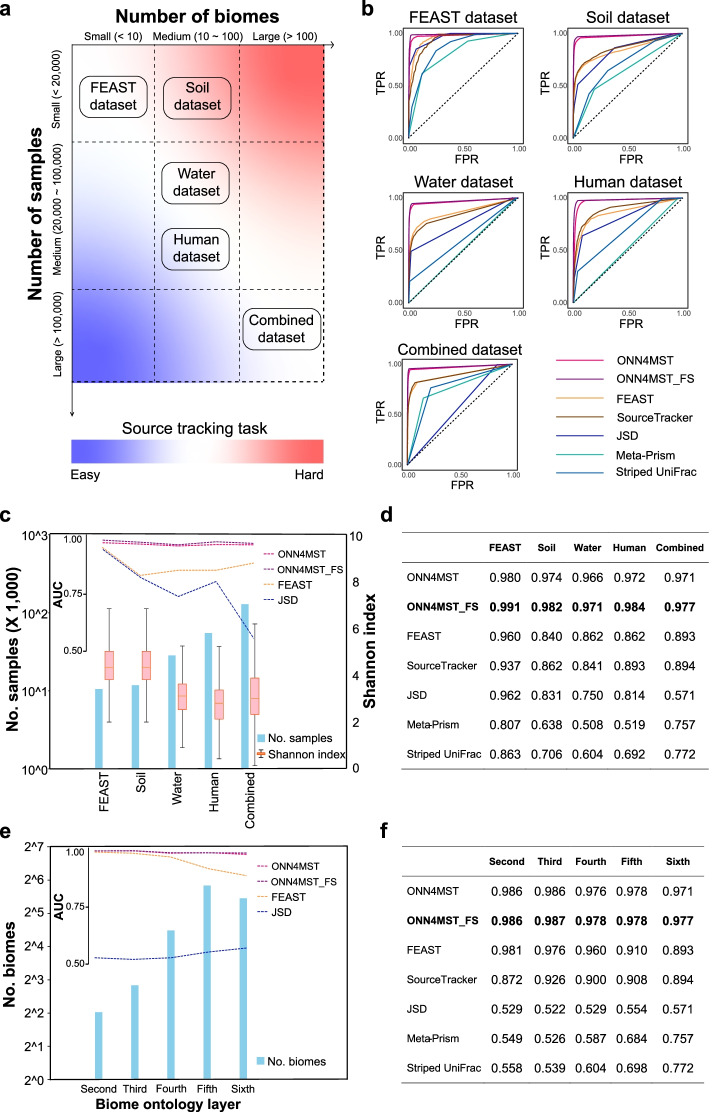
ONN4MST’s prediction accuracies are among the best on different datasets and different biome layers, while the performance of ONN4MST does not depend heavily on the number of biomes or number of samples in the dataset. **a** The five datasets (Combined, Human, Water, Soil, and FEAST datasets) with varied complexities have provided source tracking tasks with different difficulties. The complexity of the dataset is positively associated with the number of biomes and Shannon diversity and negatively associated with the number of samples. For example, source tracking tasks on the Soil dataset is difficult because of the medium number of biomes and small number of samples in the Soil dataset. **b** The ROC curve of ONN4MST and other methods on all five datasets. **c** The number of samples, the Shannon diversity and the source tracking results by different methods for the five datasets. The samples involved in each dataset are shown with blue bars, the Shannon diversity of each dataset is shown with red boxes, the AUC of several methods on each dataset is shown with dash lines. **d** The AUC of all methods on all five datasets. **e** The number of biomes and the source tracking results by different methods at different layers for the Combined dataset. The samples involved in each biome ontology layer are shown with blue bars, the AUC of different methods on each layer is shown with dash lines. **f** The AUC of all methods at different layers. Abbreviations: ONN4MST_FS, ONN4MST using selected features

We constructed five datasets, representing sample collections with different numbers of biomes and samples, covering more than 100,000 real microbial community samples (Additional file 1: Table S1 and Table S2). These five datasets contain samples from different niches including “Host_associated,” “Environmental,” and “Engineered” as top biomes, which are representatives of high-quality microbial community samples in public resources (Additional file 1: Table S2, Methods). Since these five datasets were designed to have varied complexities, each including a different number of samples from a different number of biomes, they could serve well for the evaluation of ONN4MST and other methods (Fig. 2a): The Combined dataset contains 125,823 samples and 114 biomes, which represents the largest dataset, as well as the largest model (the general model), used in this study. The FEAST dataset contains only 10,270 samples and 3 biomes. While the Human dataset, Water dataset, Soil datasets are respectively with moderate sample sizes (Additional file 1: Table S1).

First and foremost, ONN4MST’s performances on all five datasets were evaluated. Regardless of the datasets used for evaluation, the predicted biomes (i.e., biomes with dominant contribution quantified by ONN4MST) were very close to the actual biomes in most cases. For example, ONN4MST could achieve an accuracy of 0.99 and AUC of 0.97 on searching the Combined dataset with 125,823 samples from 114 biomes. When we applied ONN4MST on Human, Soil, Water, and FEAST datasets, the accuracy and AUC of ONN4MST were also higher than 0.98 and 0.96 for these datasets (Table 1, Additional file 1: Fig. S3).

**Table 1:** Evaluation of ONN4MST on all five datasets

Dataset	No. biomes	No. samples	All features					Selected features				
			Pr	Rc	Acc	*F*_max_	AUC	Pr	Rc	Acc	*F*_max_	AUC
Combined	114	125,823	0.826	0.662	0.995	0.740	0.971	0.868	0.774	0.997	0.820	0.977
Human	25	53,553	0.822	0.521	0.984	0.695	0.972	0.894	0.826	0.991	0.863	0.984
Water	44	27,667	0.842	0.766	0.992	0.803	0.966	0.854	0.764	0.992	0.813	0.971
Soil	16	11,528	0.915	0.778	0.986	0.850	0.974	0.892	0.881	0.989	0.890	0.982
FEAST	3	10,270	0.793	0.795	0.984	0.803	0.980	0.895	0.812	0.989	0.862	0.991

ONN4MST achieved the accuracy higher than 0.98 for all five datasets, and the AUC higher than 0.97 for all five datasets. For each dataset, we used the model trained on that dataset for evaluation. The evaluation procedure of the ONN model is described in the “Methods” section. ONN4MST based on all features and selected features were both evaluated at the bottom (sixth) layer with a threshold of 0.5

*Abbreviations*: *Pr* precision, *Rc* recall, *Acc* accuracy

ONN4MST based on selected features performed equally well or better than that based on all features. We conducted feature selection by using a random forest model, and 1462 features (taxa) were selected from all 44,668 features. ONN4MST uses a total of 44,668 features, but ONN4MST_FS only uses 1462 selected features (see the “Methods” section and Additional file 2). Results showed that based on 1462 selected features, ONN4MST_FS could attain slightly higher accuracy (0.997 vs. 0.995, on Combined dataset), AUC and *F*_max_ compared to ONN4MST using all features (Table 1, Additional file 1: Fig. S3), which means that there is a certain degree of redundancy among all 44,668 features, and we can achieve the same accuracy with just 1462 features compared with that using all 44,668 features. These results have emphasized the scalability and stability of the general model built based on the Combined dataset, either based on using all features, or using selected features.

Furthermore, we evaluated the universality of the general model built based on the Combined dataset, by applying it directly on the Human, Water, Soil, and FEAST datasets. It was found that the source tracking by using the general model was successful on those datasets which are composed of samples mostly from the Combined dataset’s samples (Additional file 1: Table S3, results on Human, Water, Soil datasets). However, when we applied the general model on datasets in which most of the samples were not previously observed in the general model or have more detailed biome ontology compared to the biome ontology used in the general model, the general model would not perform well (Additional file 1: Table S3, results on FEAST dataset). Besides, results showed that it was unsuccessful when we applied the human model (the model built based on the Human dataset) for source tracking on Soil and Water datasets (Additional file 1: Table S4).

Additionally, we have built a simple neural network without ontology structure, and evaluated the simple neural network on all five datasets. Results showed that the ONN model benefits a lot in accuracy and generalization by encoding ontology structure into neural network. For example, on the FEAST dataset, the evaluation results based on all features show that the AUC achieved by the simple neural network and the ONN are 0.890 and 0.980, respectively (Table 1 and Additional file 1: Table S5). 

### Comparison of ONN4MST and other source tracking methods

We then compared all six source tracking methods on all five datasets with different complexities (Fig. 2a). Results on all five datasets were evaluated separately (Fig. 2b,d). Among the four datasets excluding the FEAST dataset, ONN4MST was superior to other methods: ONN4MST reached an AUC of 0.97, while other methods only reached a maximum of 0.89 (Fig. 2d). As for the FEAST dataset, ONN4MST reached an AUC of 0.99, while other methods also reached a maximum of 0.96.

The performances of ONN4MST on five datasets are not sensitive to the complexities of datasets (Fig. 2c). The complexity of the dataset is positively associated with the number of biomes and Shannon diversity and negatively associated with the number of samples. And the five datasets we have used have different complexities (Fig. 2a,c). ONN4MST achieved robust performances with AUC > 0.96 on all five datasets. While other methods, such as FEAST and JSD, are sensitive to the complexities of datasets. For example, the Soil dataset is among those with the highest Shannon diversity, and the AUC of the FEAST method (Fig. 2c, orange dash line) on the Soil dataset is lower than those on the Water, Human, and Combined datasets. The high AUC on the FEAST dataset is mainly due to the small number of biomes used in the FEAST dataset (Additional file 1: Table S1). On the other hand, the performance of ONN4MST on each dataset did not depend heavily on the number of samples in that dataset (provided that there are at least 10,000 samples in the dataset) (Fig. 2c). Furthermore, the prediction accuracies were not biased for certain biomes (provided that there are at least 100 samples in each biome) (Additional file 1: Table S6).

We further analyzed ONN4MST’s performances at different biome layers (Fig. 2e,f). Since it is the only method available that could source track samples at different layers of biome ontology, we have remolded other methods’ search scheme into a hierarchical prediction scheme (see the “Methods” section), so that their results are comparable to ONN4MST’s. Results have clearly shown that ONN4MST and ONN4MST_FS reached an AUC of 0.97 in minimum at all layers for the Combined dataset and these were noticeably superior to other methods (Fig. 2e,f). Thus, ONN4MST is not just the only method available that could source track at different layers, but also the best method even when other methods were remolded for such purpose.

### Running time and memory utilization benchmark

We evaluated the time and memory cost of all methods using a computational platform comprising Intel(R) Xeon(R) CPU E7-4809 v3 @ 2.00GHz CPU (64 cores in total) with 315 GB RAM, Nvidia Tesla K80 GPU with 12 GB RAM. For time cost comparison, all actual times (search time, excluding I/O time) were converted to the equivalent time on a single core.

**Fig. 3:**
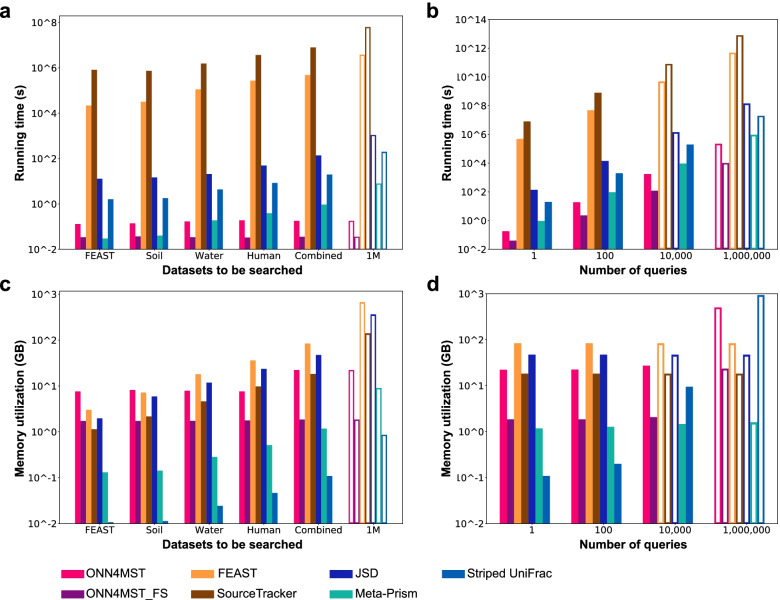
ONN4MST is superior to other methods in search time and memory utilization. **a** Running time of different methods when search one query against different datasets. **b** Running time of different methods when search queries of different sizes against Combined dataset. **c** Memory utilization of all methods when search one query against different datasets. **d** Memory utilization of all methods when search queries of different sizes against Combined dataset. Note: a hollow bar means that the value represent by this bar is the result of linearly extrapolation, both for running time and for memory utilization. Abbreviations: ONN4MST_FS, ONN4MST using selected features; 1M, results of linearly extrapolation with one million samples in use

ONN4MST is superior to other methods in search time and memory utilization where the superiority expands as the number of source samples increases (Fig. 3). First of all, we tested the time cost by searching a single query against the five datasets respectively. For the Combined dataset including 125,823 source samples, ONN4MST and ONN4MST_FS took 0.18 s and 0.04 s, respectively, while distance-based methods took at least 1 s for a query. And FEAST took more than 100,000 s, and SourceTracker took even more time (Fig. 3a, on the Combined dataset, as also verified in Shenhav et al. [12]). Interestingly, though the time spent by FEAST and Source Tracker per thousand of source samples were both less than those reported in Shenhav et al. [12], these two methods costed magnitudes more time than ONN4MST (Fig. 3a). When we linearly extrapolated the number of source samples to one million in the dataset to be searched, the advantage of ONN4MST over other methods still held (Fig. 3a, hollow bars). When searching a different number of queries against the Combined dataset, we observed the time cost follows this trend: supervised methods (ONN4MST and ONN4MST_FS) ≤ distance-based methods (JSD, Meta-Prism and Striped UniFrac) < unsupervised methods (FEAST and SourceTracker) (Fig. 3b). Again, when we linearly extrapolated the number of queries to one million in a batch, the advantage of ONN4MST over other methods still held (Fig. 3b, hollow bars).

When memory utilization was evaluated, we have also observed the superiority of ONN4MST over most of the other methods. Specifically, when searching a single query against the Combined dataset, ONN4MST and ONN4MST_FS needed 22 GB and 2 GB of memory, respectively; while FEAST and SourceTracker needed 84 GB and 18 GB of memory, respectively; and JSD needed 47 GB of memory. Striped UniFrac and Meta-Prism (https://github.com/HUST-NingKang-Lab/Meta-Prism-2.0) were comparable with ONN4MST_FS in memory utilization, since they have optimized the data structure for sample comparison. When the number of queries in a batch exceeded 10,000, or the size of the dataset to be searched varies, ONN4MST and ONN4MST_FS remain the ones that needed the least memory (Fig. 3c,d). Details about running time and memory utilization are presented in Additional file 1: Tables S7-S10.

### Utility of ONN4MST in various source tracking applications

The objective of microbial community sample source tracking is knowledge discovery from the huge amount of microbial community samples of heterogeneous sources. Thus, we showcased the ability of ONN4MST in knowledge discovery from several perspectives: firstly, it can ensure accurate and interpretable source tracking, even on distinguishing samples from ontologically-close biomes; secondly, when samples’ biomes are previously less studied or unknown, ONN4MST could provide accurate and interpretable clues for possible biome at higher layers, supplementing the information about such less studied biome; thirdly, ONN4MST could help for accurate microbial contaminant detection; finally, “open search” of sample among the source samples with almost all possible biomes could identify similar samples from ontologically-remote biomes, leading to novel knowledge discovery.

### Centenarians share similar gut microbiota with young individuals

ONN4MST can distinguish samples from ontologically-close biomes, thus offers a quantitative way to characterize the development of human gut microbial community. In this context, we leveraged external sources of young individuals (30 years old on average) to understand the unique properties of gut microbiota in centenarians (persons over 100 years old). To demonstrate this capability, we first built a self-defined ONN model with two layers of biome ontology: “human gut” as the first layer, while “Young human gut” and “Others or unknown” at the second layer, through using a training set which contains 5000 randomly selected human gut samples from the Combined dataset (Additional file 1: Table S1), together with 800 randomly selected human gut samples from young individuals in published studies [22, 23]. Then, samples from centenarians (30 from Italy, and 51 from China) [22, 23] were used as queries for performing source tracking with the self-defined ONN model. Results revealed a significantly larger “Young human gut” contribution (Wilcoxon-test, *p* < 1e-3) in centenarians (Additional file 1: Fig. S4), regardless of the locations where these samples were collected, which were consistent with the results of published studies [22, 23]. We further tested whether these profiles are selective to centenarians but not normal seniors. We collected 770 samples of normal seniors from another published study [28] as queries for comparison. However, we were unable to detect a significant “Young human gut” contribution in these normal seniors (Additional file 1: Fig. S4). Therefore, we demonstrate that the gut microbiome of centenarians differs from that of normal seniors and shows a youthful pattern.

ONN4MST can also help for inferring niche association in the human microbiome. The niche association analysis in a previous study has shown that there are body site-specific subspecies clades [29]. Here, we used ONN4MST to explore the association of niche and microbes based on 303 samples targeting diverse body sites from the EBI MGnify database [8] (Study MGYS00001056). These 303 samples belong to three human body sites, including 90 gut samples, 183 oral samples, and 30 vaginal samples. Notably, these samples are not included in the Combined dataset. We used the ONN4MST model to predict the source of these samples. Results showed that ONN4MST could identify these samples to the actual biome (i.e., “Host_associated”) at the second layer. The prediction accuracies reasonably decreased when it comes to biome layer three, layer four and layer five. And about half of the oral sample were misclassified at the fifth layer, while all the vaginal samples were misclassified at the fourth and fifth layers (Additional file 1: Table S11). We further investigated these misclassified oral and vaginal samples, and found that about half of the oral samples were classified as the biome “Large_intestine” at fifth layer, while the vaginal samples were classified as the biome “Skin” at fourth layer. These investigations demonstrated that oral has non-neglected niche association with gut, while vagina has strong niche association with skin.

### Detecting microbial contamination in built environment

**Fig. 4:**
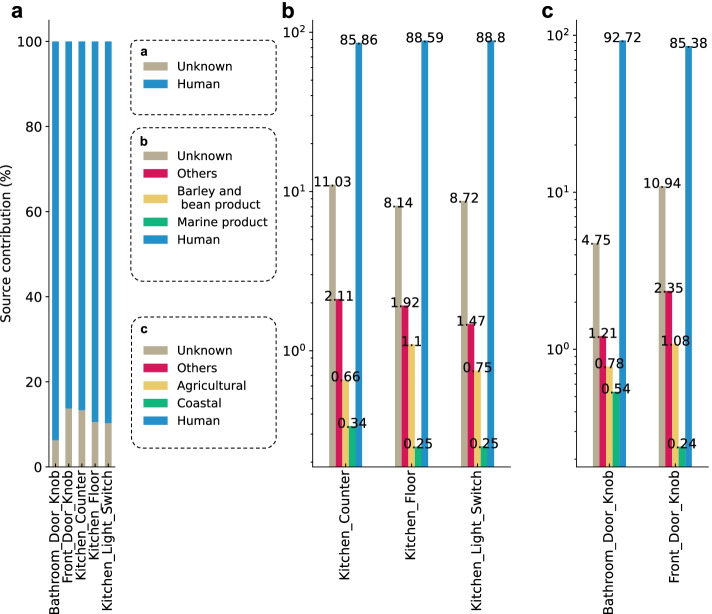
The contribution of the unknown sources in indoor house surface samples using ONN4MST. **a** Mean source contributions considering 4 human skin sources (hand, foot, nose, and skin—other across all inhabitants) using data from Lax et al*.* [24]. **b**, **c** Further decomposition of the unknown sources existed in **a** has revealed other microbial contaminates in built environment

To validate ONN4MST's ability on microbial contamination detection, we analyzed microbial community data collected by Lax *et al.* [24] In this analysis, we investigated microbial contamination at several indoor house surfaces. We used skin samples from several body parts (skin, foot, hand, and nose) and additional environmental, plants, and mammal samples from the Combined dataset (Additional file 1: Table S1) as source samples, and samples from indoor house surfaces (“Bathroom Door Knob”, “Front Door Knob”, “Kitchen Counter”, “Kitchen Floor”, and “Kitchen Light Switch”) as queries. Our analysis results by using ONN4MST have shown that microbial communities on these surfaces mostly originated from humans (Fig. 4a), largely in agreement with the original analyses of Lax et al. [24] using SourceTracker, and differs slightly from the results of Shenhav et al*.* [12] These results were reasonable considering the strong influence of skin microbial communities on indoor house surfaces [30], while they have again emphasized the challenge of source disambiguation for methods that do not consider ontology structure of the biomes. That is, treating each individual sample as an independent potential source would make differentiation of tiny sample differences among ontologically-close biomes impossible, thus underestimating the contributions of known sources at higher layers. We further investigated the composition of the human and unknown sources existed in Fig. 4a. In addition to the contribution of human, we found evidence for contributions from mammals (0.1–1.7%), soils (0.1–3.1%), barley and bean product (0.6–1.1%), and marine product (0.2–0.4%) for kitchen environments, and potential evidence for contributions from agricultural (0.7-1.1%) and coastal (0.2–0.6%) for door knobs, were also identified (Fig. 4b,c, Additional file 1: Table S12).

### Source tracking of environmental samples from less studied biomes

**Fig. 5:**
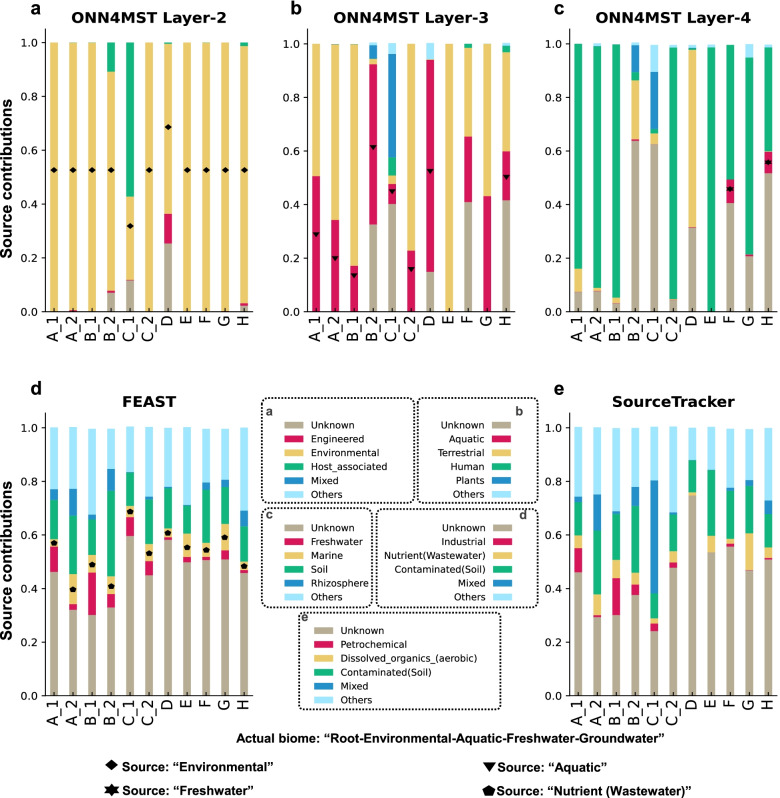
Successful source tracking of environmental samples from a less studied biome by using ONN4MST. Results were based on using 11 samples from groundwater environment, which represented a biome previously less studied. **a–c** Source tracking results by using ONN4MST at the second, third, and fourth layers. **d** Source tracking results by using FEAST. **e** Source tracking results by using SourceTracker. Actual biome of query sample: “Root-Environmental-Aquatic-Freshwater-Groundwater.” A_1, A_2: two samples collected from a single well; B_1, B_2: two samples collected from another single well; C_1, C_2: two samples collected from the third single well; **D-H**: samples collected from other five wells, respectively

This investigation was based on searching 11 groundwater samples from another published study [25] (the biome “Groundwater” is less studied, with a handful of samples in the MGnify database, Additional file 1: Table S2) against the Combined dataset. ONN4MST could identify significantly larger proportions of inputs from biome “Aquatic” (average contribution 0.32, Fig. 5b) compared with “Marine” (average contribution 0.09, Wilcoxon-test, *p* = 0.007, Fig. 5c), coupled with considerable inputs from “Terrestrial” (average contribution 0.46, Fig. 5b,c), suggesting that the samples might be collected from terrestrial water (i.e., river, lake, groundwater), or their sediment, rather than water from marine (ocean or sea). Notably, for these “Groundwater” samples, FEAST and SourceTracker assigned a large proportion of “Unknown” (Fig. 5d,e). Such differences in quantification are mainly due to the fact that ONN4MST could screen the whole biome ontology, and quantify contributions at different layers, enabling it to at least tell the potential inputs at a lower resolution but with higher fidelity. Whereas FEAST and SourceTracker were designed without considering the biome ontology, they would assign "Unknown" for many of these samples. Additionally, the increasing unknown sources’ contribution from the second layer to the sixth layer (Fig. 5a–c, Additional file 1: Fig. S5), as well as the large proportion of unknown sources’ contributions quantified by FEAST and SourceTracker (Fig. 5d,e), also suggest that there is indeed a certain degree of microbial dark matters remain to be discovered. Although ONN4MST may have limitations in detecting microbial community samples with biomes of mixed backgrounds, especially when the number of training samples is few in a specific biome, or when samples are from biome less studied, ONN4MST could provide interpretable clues for possible biome at higher layers in the biome ontology, which could be useful in guiding the manual curation of these samples.

We also evaluated ONN4MST for samples from biomes not present in the ONN4MST model. We collected 148 samples from the ceca of birds in the EBI MGnify database [8] (Study MGYS00005593). Notably, “Bird” represents a newly introduced biome (not included in the biome ontology when generating the ONN4MST model) which belongs to the biome of “Host_associated,” and these 148 samples from ceca of bird are not in the Combined dataset. We used the ONN4MST model to predict the source of these ceca samples from birds. Results showed that ONN4MST identified these ceca samples as from the biome of “Host_associated” at the second layer of biome ontology, yet from “Human” and “Mammals” at the third layer of biome ontology (Additional file 1: Table S13). We should emphasize that although ONN4MST is unable to predict the actual biome of samples from biomes which are not present in the training model, ONN4MST could provide interpretable clues for possible biome at higher layers in the biome ontology, which could be useful in guiding the manual curation of these samples.

We further evaluated ONN4MST for samples from the same biome but with different characteristics. Previous studies have reported that microbial community samples from the soil with different characteristics possessed high diversity [31]. To evaluate the capability of ONN4MST for predicting samples from the same biome with different characteristics, we introduced another cohort, about the seasonal changes of the Hadza people’s gut microbial communities [26]. In this evaluation, 203 gut microbiome samples of the Hadza hunter-gatherers of Tanzania were used for source tracking. These 203 gut microbiome samples are divided into “Dry” and “Wet” categories, in which 106 samples are from the “Dry” category meaning samples are collected from humans in dry seasons, and the other 97 samples are from “Wet” category meaning samples are collected from human in wet seasons. Results showed ONN4MST could classify the majority of these samples from human gut, with a few samples misclassified (Additional file 1: Table S14). However, the proportion of samples misclassified were higher in “Wet” category than in “Dry” category. These findings suggest that ONN4MST predictions are influenced by the confounding factors such as seasons in which the samples were collected. This is reasonable, since in different seasons, the Hadza people’s diets are drastically different, and the diets could asset strong influence on gut microbial communities.

### Discovery of similar samples from ontologically-remote biomes

Another advantage of ONN4MST in source tracking is its ability for “open search” without any *a priori* knowledge about possible biomes where the query might be from, enabling it for novel knowledge discovery. We tested ONN4MST’s “open search” results and found that it could discover similar samples among ontologically-remote biomes “Engineered,” “Host_associated,” and “Environmental” (Additional file 1: Table S15). While some of the samples from the biome “Root-Environmental-Aquatic-Marine-Intertidal_zone” share similar environments (Baltic Sea) with the query sample from the biome “Root-Engineered-Wastewater-Industrial_wastewater-Petrochemical,” the literature has also verified that this query sample was marine-sourced “MGYS00005175” (from MGnify database [8]). Such examples were plentiful (Additional file 1: Fig. S6), and many had very high contributions (Additional file 1: Table S15). However, there were also examples which might indicate possible mis-annotation or possible contaminations of samples in the MGnify database [8]. For instance, more than 10 samples from the study “MGYS00001610” (from MGnify database [8]) with annotated biome “Root-Engineered-Wastewater-Water_and_sludge” have large proportion of contributions from biome “Root-Host_associated-Mammals-Digestive_system-Large_intestine-Fecal” (Additional file 1: Fig. S6), while Lin et al. [32] has also verified that these samples were collected from biogas of digested swine manure. These results have verified our hypothesis that open search of sample among the source samples with almost all possible biomes could reveal remotely-similar samples, leading to novel knowledge that is never identified or interpreted before.

## Discussion

ONN4MST was designed to address the urgent need for fast, accurate and interpretable microbial community source tracking. It has been built based on an ONN model, which has provided a solution for source tracking among sub-million samples and hundreds of biomes, outperforming state-of-the-art methods, thus enabling knowledge discovery from these heterogeneous samples. Microbial community sample source tracking has become increasingly important, mainly due to the needs of source tracking in multiple areas. The requirements for high accuracy, high speed, and high interpretability have thus become critical considerations for a successful source tracking method, especially when faced with the ever more complex situation where sub-million microbial community samples from hundreds of biomes are provided as possible sources for search.

The superiority of ONN4MST is established in several contexts. Firstly, ONN4MST is very robust against dataset heterogeneity: from a dataset with the number of biomes ranging from a handful to more than a hundred, as well as with the number of samples ranging from a few thousand to sub-million, it always provides the highest accuracies (AUC > 0.97) among state-of-the-art methods compared, making it the most scalable source tracking method. Secondly, based on the Human, Water and Soil datasets, the source tracking accuracies are all near-perfect (AUC > 0.97), indicating that ONN4MST could provide reliable insights for downstream analysis on implicating taxonomic or functional differences between healthy and diseased phenotypes, or on illuminating tiny differences among environmental samples from even slightly different niches. Furthermore, ONN4MST is very efficient as regard to speed and memory usage. For example, when source tracking one hundred samples against a database of sub-million samples (i.e., the Combined dataset) on a standard computational platform (see the “Methods” section for details), ONN4MST would take about 20 s and a memory usage of 22GB during the search process, while FEAST method would take many days and a memory usage of 84 GB. The time usage and memory usage of ONN4MST is several orders of magnitude smaller than FEAST method. Finally, the ability of ONN4MST for ‘open search’, without any a priori knowledge about possible biomes where the query might be from, enables it for interpretable knowledge discovery.

The advantage of ONN4MST over other state-of-the-art source tracking methods is essentially dependent on two technical advancements: the deep learning model, and the ontology structure. Though the currently ongoing shift towards supervised learning methods is not surprising for the source tracking research, the superior performance of ONN4MST over existing methods is still quite pronounced. ONN4MST’s advantage also stems from its consideration of the ontology structure of the biomes: by embedding the ontology considerations into the deep learning model, ONN4MST naturally becomes suitable for solving the ontology relationships among biomes. Taken together, ONN4MST is a strong complement to existing methods, as it could be very helpful and quick and perhaps be useful in determining the unknowns that are high with FEAST and SourceTracker.

ONN4MST is not without limitations. Most importantly, the accuracy of ONN4MST is heavily dependent on the ONN model built based on existing biome ontology information. If there comes a new biome ontology with more detailed biomes involved (for example, if we need to refine the source tracking results to human gut down, to differentiate niches such as adult’s gut from infant’s gut), or simply with more biome relationships involved, then the ONN model should be re-trained for accurate source tracking. Such biome ontology-wide scalability problem could potentially be solved by transfer learning approaches.

## Conclusions

In summary, ONN4MST is an ontology-aware deep learning method that has further improved microbial source tracking, enabling highly accurate, ultrafast and interpretable source tracking against large-scale microbial community samples. ONN4MST has enabled in-depth pattern and function discoveries among sub-million microbial community samples, allowing for tracking the potential origin of microbial communities with diverse niche backgrounds, as well as distinguishing samples from different health conditions or diverse environments. Thus, it could have a broader area of application, such as contamination screening, novel or refined biome discovery, new functional microbiome discovery, and even source tracking of biomes from which protein sequences could be supplemented for computational protein 3D structure prediction [33, 34].

## Supplementary Information

**Additional file 1: Table S1.** Samples and data used for model building and testing. **Table S2.** Biomes and number of samples used in EBI MGnify and this study. **Table S3.** Evaluation of ONN4MST using the general model built based on the combined dataset. **Table S4.** Evaluation of ONN4MST using the model trained on the human dataset. **Table S5.** Evaluation of simple neural network on all five datasets. **Table S6.** Results of five biome from “Human” using all features by ONN4MST at fifth layer. **Table S7.** Running time when performing source tracking with one query against different datasets. **Table S8.** Running time when performing source tracking with different sizes of testing sets on combined dataset. **Table S9.** Memory utilization when performing source tracking with one query against different datasets. **Table S10.** Memory utilization when performing source tracking with different sizes of testing sets on combined dataset. **Table S11.** The prediction results 303 samples from diverse human body sites. **Table S12.** Average source contributions from mammals (pets) and soil for indoor house environments. **Table S13.** The prediction results of 148 samples from ceca of bird. **Table S14.** The prediction results for 203 gut microbiome samples of the Hadza hunter-gatherers of Tanzania. **Table S15.** The open searching results by using ONN4MST against the combined dataset. **Table S16.** Databases and software parameters used in this study. **Figure S1.** The architecture of the ONN model. **Figure S2.** Overview of ONN4MST for microbial source tracking. **Figure S3.** ROC curves of ONN4MST on all five datasets. **Figure S4.** ONN4MST estimations of source contribution to centenarians’ gut microbiome. **Figure S5.** Source tracking results of a less studied biome. **Figure S6.** Knowledge discovery of similar samples from ontologically-remote biomes.

**Additional file 2.** The features used in ONN4MST and the selected features used in ONN4MST_FS. There are 44,668 taxa (or features) in total used in ONN4MST, while ONN4MST_FS (ONN4MST based on selected features) has utilized only 1,462 selected features.

**Additional file 3.** Supplementary method about the data representation and other source tracking methods used in this study.

**Additional file 4.** Data download links for all five datasets used in this study.

## Data Availability

The selected samples from the Combined dataset, which were assigned to Human dataset, Water dataset, and Soil dataset, respectively, were annotated with their respective assignments in Additional file 1: Table S2. Data download links are provided in Additional file 4. The dataset used in the case study of centenarian was collected and studied by Bian et al. [22] (accession number SRP107602) and Biagi et al. [23] (from multiple sources). The dataset used in the case study of exploring the association of niche and microbes was from the EBI MGnify database [8] (Study MGYS00001056). The dataset used in the case study of detecting microbial contamination in built environment was collected and studied by Lax et al. [24] (accession number ERP005806). The dataset used in the case study of less studied biomes was collected and studied by Alsalah et al. [25] (accession number PRJEB9501). The dataset used in the case study of the bird biome was from the EBI MGnify database [8] (Study MGYS00005593). The dataset used in the case study of Hadza people’s gut microbial communities was collected and studied by Samuel et al. [26] (accession number PRJNA392012, PRJNA392180). All source codes have been uploaded to the website at: https://github.com/HUST-NingKang-Lab/ONN4MST [35]. Detailed parameters of software and package we used in this study are provided in Additional file 1: Table S16. All datasets used in this study are publicly available.

## References

[1] Turnbaugh PJ, Ley RE, Hamady M, Fraser-Liggett CM, Knight R, Gordon JI (2007). The human microbiome project. Nature..

[2] Proctor LM, Creasy HH, Fettweis JM, Lloyd-Price J, Mahurkar A, Zhou W (2019). The Integrative Human Microbiome Project. Nature..

[3] Gilbert JA, Jansson JK, Knight R (2014). The Earth Microbiome project: successes and aspirations. BMC Biol.

[4] Thompson LR, Sanders JG, McDonald D, Amir A, Ladau J, Locey KJ (2017). A communal catalogue reveals Earth's multiscale microbial diversity. Nature..

[5] Dominguez-Bello MG, De Jesus-Laboy KM, Shen N, Cox LM, Amir A, Gonzalez A (2016). Partial restoration of the microbiota of cesarean-born infants via vaginal microbial transfer. Nat Med.

[6] Thomas S, Izard J, Walsh E, Batich K, Chongsathidkiet P, Clarke G (2017). The host microbiome regulates and maintains human health: a primer and perspective for non-microbiologists. Cancer Res.

[7] Tokeshi M (1993). Species abundance patterns and community structure. Adv Ecol Res.

[8] Mitchell AL, Almeida A, Beracochea M, Boland M, Burgin J, Cochrane G (2019). MGnify: the microbiome analysis resource in 2020. Nucleic Acids Res.

[9] Mukherjee S, Stamatis D, Bertsch J, Ovchinnikova G, Sundaramurthi Jagadish C, Lee J (2021). Genomes OnLine Database (GOLD) v.8: overview and updates. Nucleic Acids Res.

[10] Lladó S, López-Mondéjar R, Baldrian P (2018). Drivers of microbial community structure in forest soils. Appl Microbiol Biotechnol.

[11] Grond K, Guilani H, Hird SM (2020). Spatial heterogeneity of the shorebird gastrointestinal microbiome. R Soc Open Sci.

[12] Shenhav L, Thompson M, Joseph TA, Briscoe L, Furman O, Bogumil D (2019). FEAST: fast expectation-maximization for microbial source tracking. Nat Methods.

[13] Simpson JM, Santo Domingo JW, Reasoner DJ (2002). Microbial source tracking: state of the science. Environ Sci Technol.

[14] Lozupone C, Knight R (2005). UniFrac: a new phylogenetic method for comparing microbial communities. Appl Environ Microbiol.

[15] Smith A, Sterba-Boatwright B, Mott J (2010). Novel application of a statistical technique, random forests, in a bacterial source tracking study. Water Res.

[16] Knights D, Kuczynski J, Charlson ES, Zaneveld J, Mozer MC, Collman RG (2011). Bayesian community-wide culture-independent microbial source tracking. Nat Methods.

[17] Lin J (1991). Divergence measures based on the Shannon entropy. IEEE Trans Inf Theory.

[18] Zhu M, Kang K, Ning K (2021). Meta-Prism: Ultra-fast and highly accurate microbial community structure search utilizing dual indexing and parallel computation. Brief Bioinform.

[19] McGhee JJ, Rawson N, Bailey BA, Fernandez-Guerra A, Sisk-Hackworth L, Kelley ST (2020). Meta-SourceTracker: application of Bayesian source tracking to shotgun metagenomics. PeerJ..

[20] Kahanda I, Funk C, Verspoor K, Ben-Hur A (2015). PHENOstruct: Prediction of human phenotype ontology terms using heterogeneous data sources. F1000Res..

[21] Kulmanov M, Hoehndorf R (2020). DeepPheno: Predicting single gene loss-of-function phenotypes using an ontology-aware hierarchical classifier. PLoS Comput Biol.

[22] Bian G, Gloor GB, Gong A, Jia C, Zhang W, Hu J (2017). The gut microbiota of healthy aged chinese is similar to that of the healthy young. mSphere.

[23] Biagi E, Nylund L, Candela M, Ostan R, Bucci L, Pini E (2010). Through ageing, and beyond: gut microbiota and inflammatory status in seniors and centenarians. PLoS One.

[24] Lax S, Smith DP, Hampton-Marcell J, Owens SM, Handley KM, Scott NM (2014). Longitudinal analysis of microbial interaction between humans and the indoor environment. Science..

[25] Alsalah D, Al-Jassim N, Timraz K, Hong P-Y (2015). Assessing the groundwater quality at a Saudi Arabian agricultural site and the occurrence of opportunistic pathogens on irrigated food produce. Int J Environ Res Public Health.

[26] Smits Samuel A, Leach J, Sonnenburg Erica D, Gonzalez Carlos G, Lichtman Joshua S, Reid G (2017). Seasonal cycling in the gut microbiome of the Hadza hunter-gatherers of Tanzania. Science..

[27] Abadi M, Barham P, Chen J, Chen Z, Davis A, Dean J (2016). TensorFlow: a system for large-scale machine learning. Proceedings of the 12th USENIX Conference on Operating Systems Design and Implementation.

[28] Jeffery IB, Lynch DB, O'Toole PW (2016). Composition and temporal stability of the gut microbiota in older persons. ISME J.

[29] Lloyd-Price J, Mahurkar A, Rahnavard G, Crabtree J, Orvis J, Hall AB (2017). Strains, functions and dynamics in the expanded Human Microbiome Project. Nature..

[30] Timmis K, Jebok F, Rohde M, Molinari G (2018). Microbiome Yarns: microbiome of the built environment, paranormal microbiology, and the power of single cell genomics. Microb Biotechnol.

[31] Wu J, Song C, Dubinsky EA, Stewart JR (2021). Tracking major sources of water contamination using machine learning. Front Microbiol.

[32] Lin Q, He G, Rui J, Fang X, Tao Y, Li J (2016). Microorganism-regulated mechanisms of temperature effects on the performance of anaerobic digestion. Microb Cell Factories.

[33] Ovchinnikov S, Park H, Varghese N, Huang P-S, Pavlopoulos GA, Kim DE (2017). Protein structure determination using metagenome sequence data. Science..

[34] Wang Y, Shi Q, Yang P, Zhang C, Mortuza SM, Xue Z (2019). Fueling ab initio folding with marine metagenomics enables structure and function predictions of new protein families. Genome Biol.

[35] Zha Y, Chong H, Qiu H, Kang K, Dun Y, Chen Z (2020). ONN4MST: Ontology-aware neural network for microbial community sample source tracking: GitHub.

