# Supplementary material for: Ontology-aware deep learning enables ultrafast and interpretable source tracking among sub-million microbial community samples from hundreds of niches
Source: Genome Med. 2022 Apr 26;14:43. doi: 10.1186/s13073-022-01047-5 (PMC9040266; doi:10.1186/s13073-022-01047-5)
Supplement: Supplementary file 1 — Additional file 1: Table S1. Samples and data used for model building and testing. Table S2. Biomes and number of samples used in EBI MGnify and this study. Table S3. Evaluation of ONN4MST using the general model built based on the combined dataset. Table S4. Evaluation of ONN4MST using the model trained on the human dataset. Table S5. Evaluation of simple neural network on all five datasets. Table S6. Results of five biome from “Human” using all features by ONN4MST at fifth layer. Table S7. Running time when performing source tracking with one query against different datasets. Table S8. Running time when performing source tracking with different sizes of testing sets on combined dataset. Table S9. Memory utilization when performing source tracking with one query against different datasets. Table S10. Memory utilization when performing source tracking with different sizes of testing sets on combined dataset. Table S11. The prediction results 303 samples from diverse human body sites. Table S12. Average source contributions from mammals (pets) and soil for indoor house environments. Table S13. The prediction results of 148 samples from ceca of bird. Table S14. The prediction results for 203 gut microbiome samples of the Hadza hunter-gatherers of Tanzania. Table S15. The open searching results by using ONN4MST against the combined dataset. Table S16. Databases and software parameters used in this study. Figure S1. The architecture of the ONN model. Figure S2. Overview of ONN4MST for microbial source tracking. Figure S3. ROC curves of ONN4MST on all five datasets. Figure S4. ONN4MST estimations of source contribution to centenarians’ gut microbiome. Figure S5. Source tracking results of a less studied biome. Figure S6. Knowledge discovery of similar samples from ontologically-remote biomes. [file 13073_2022_1047_MOESM1_ESM.docx]

**Additional File 1**

**Supplementary Figures and Tables**


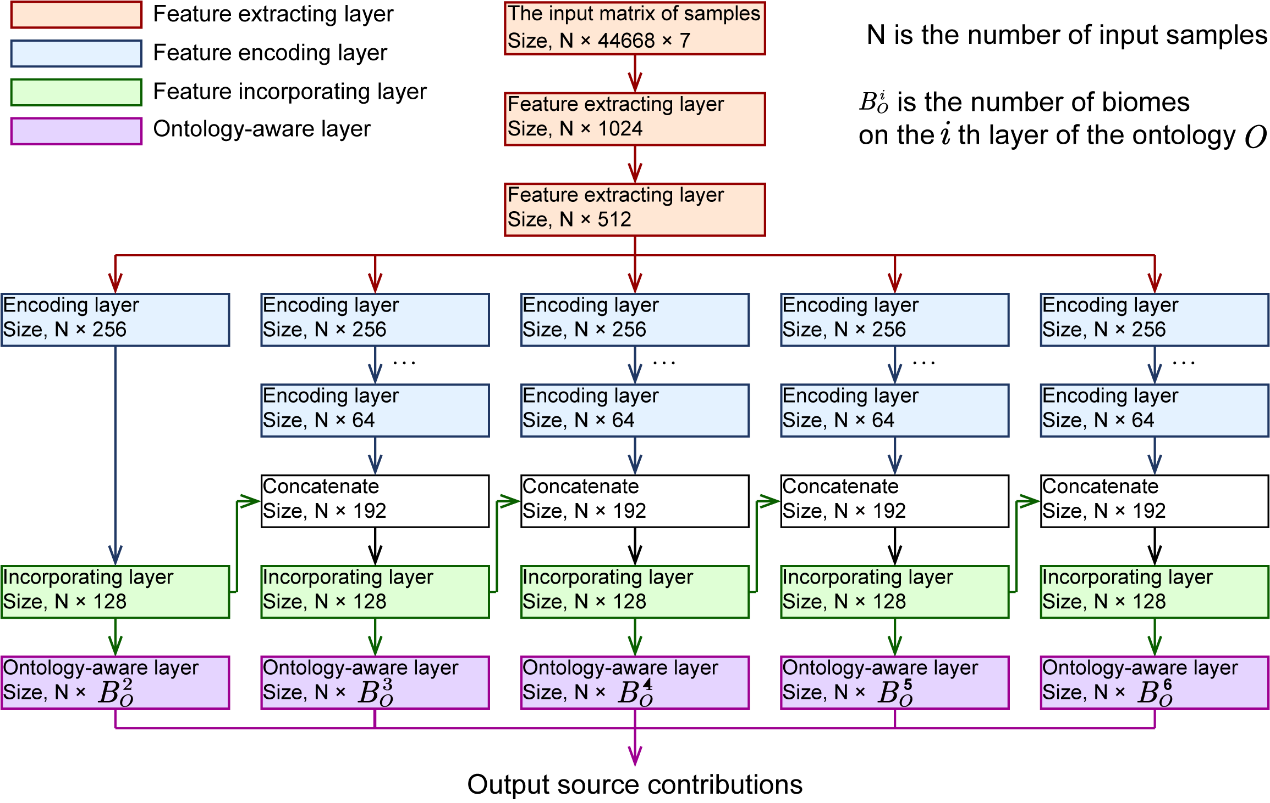


**Figure S1.** **The architecture of the ONN model**. The architecture of the Ontology-aware Neural Network could be described in four functional layers, including feature extracting layer (input layer), feature encoding layer, feature incorporating layer, and ontology-aware layer (output layer).


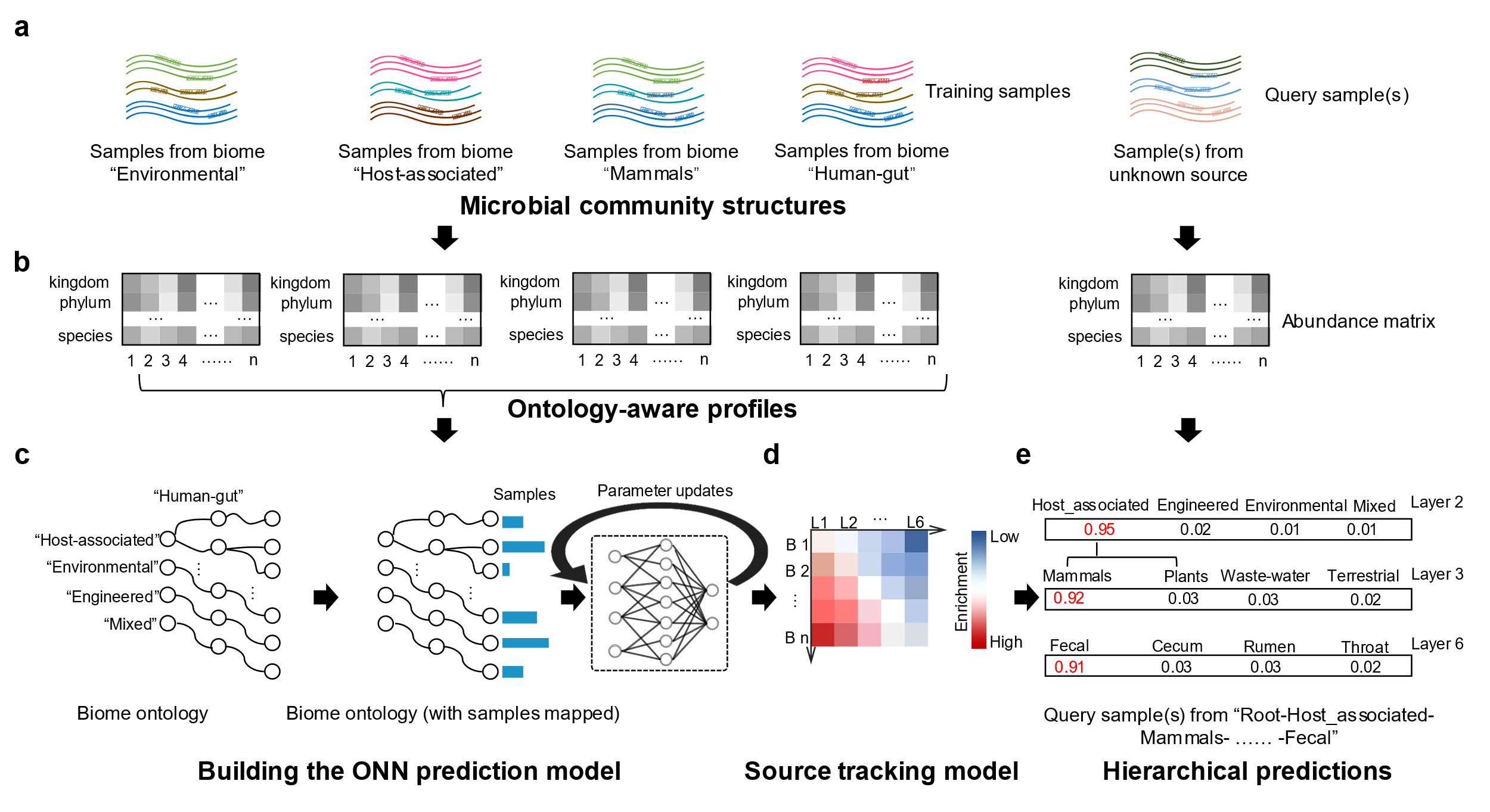


**Figure S2. Overview of using ONN4MST for microbiome sample source tracking. a.** Collecting microbial community samples; **b.** Align those samples to a phylogenetic tree to generate the Matrix as input; **c.** Building and training the ONN prediction model, the ONN prediction model was trained for 30,000 iterations or until training accuracy converged; **d.** The ONN prediction model with the highest accuracy on the training set was selected as the final well-trained ONN model; **e.** The well-trained ONN model generates a hierarchical prediction, which indicates the predicted biomes (together with contributions) of the query sample on every layers of the biome ontology.


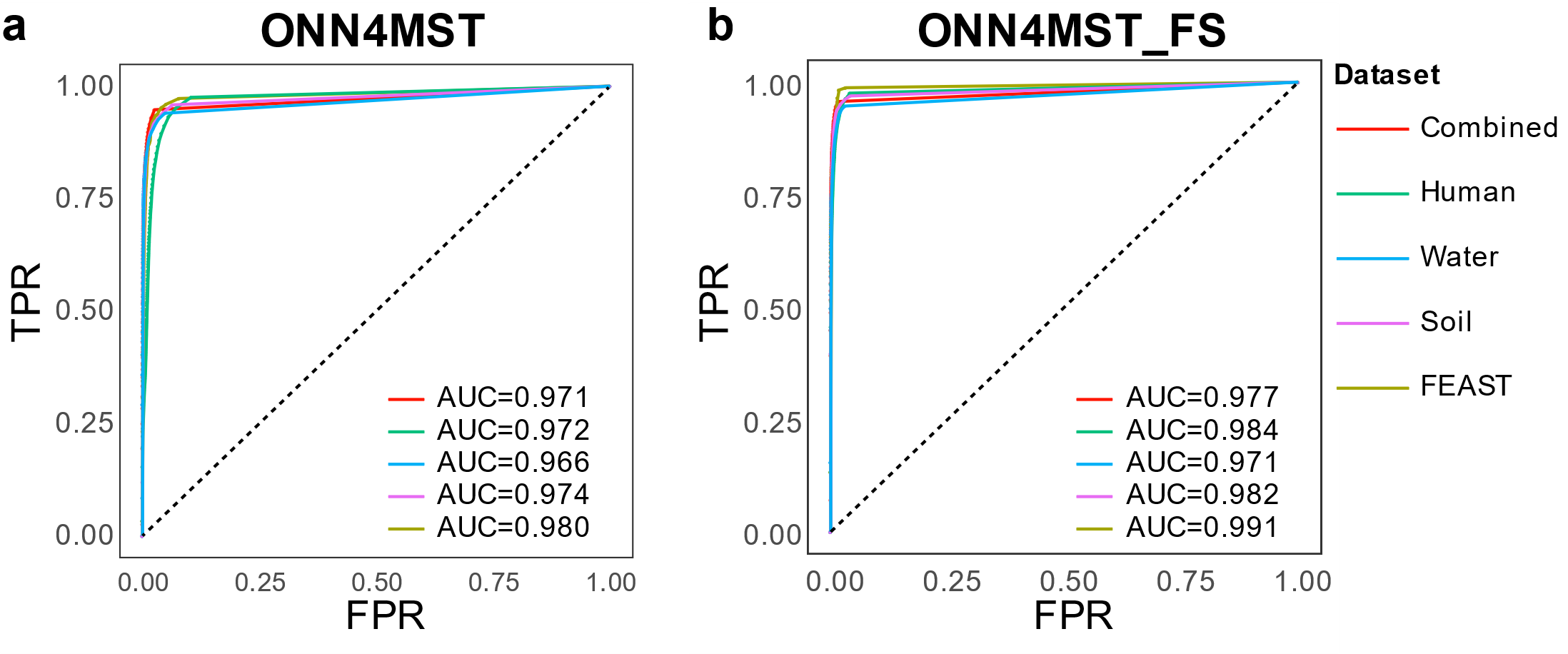


**Figure S3. ROC curves using ONN4MST on all five datasets. a.** The ROC curve of ONN4MST using all features on each dataset; **b.** The ROC of ONN4MST using selected features on each dataset; (**Abbreviations**. ONN4MST_FS: ONN4MST using selected features).


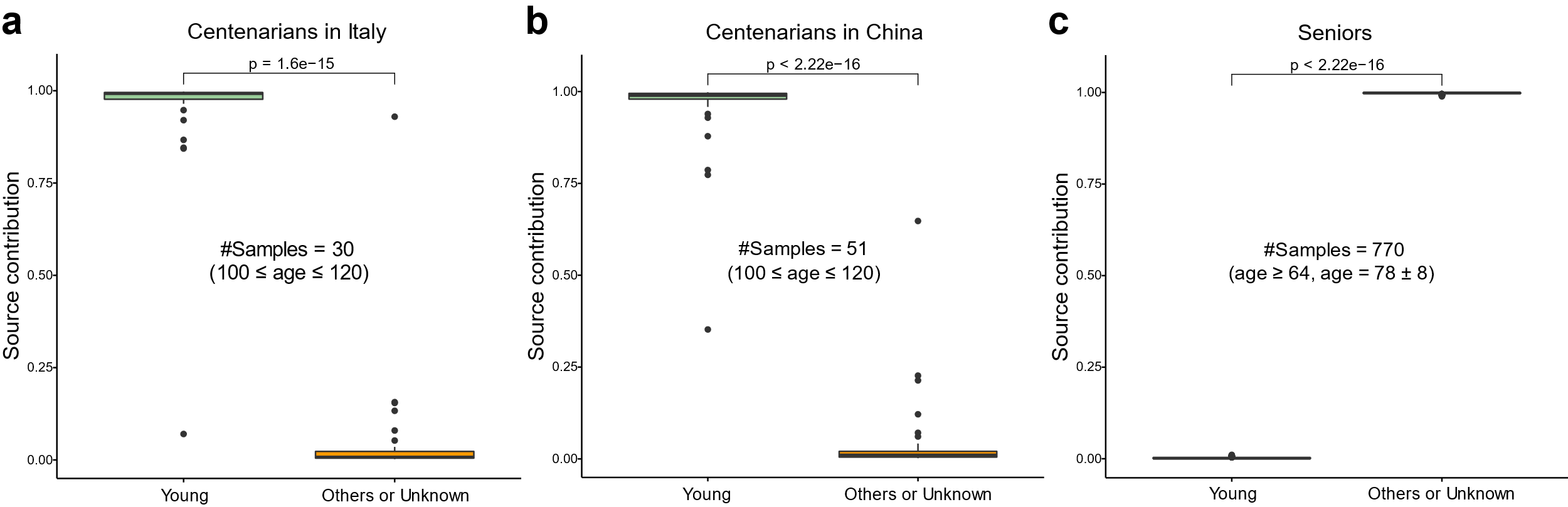


**Figure S4. ONN4MST estimations of source contribution to centenarians’ gut microbiome.** **a.** There is a significantly larger “Young human gut” contribution (Wilcoxon-test, p = 1.6e-15) in centenarians from Italy. **b.** There is a significantly larger “Young human gut” contribution (Wilcoxon-test, p <2.22e-16) in centenarians from China. **c.** A large contribution of “Unknown” is assigned in seniors (Wilcoxon-test, p < 2.22e-16).


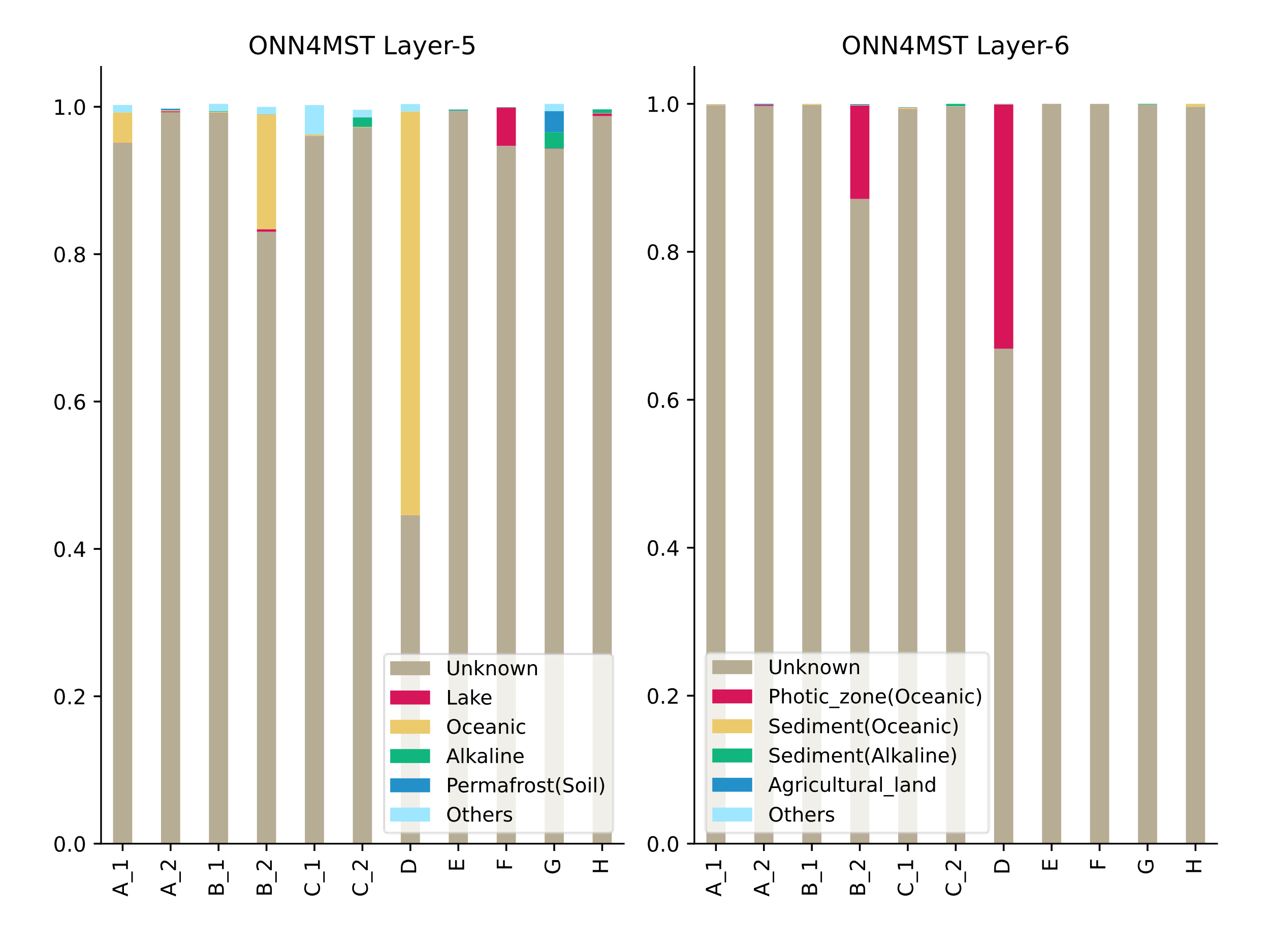


**Figure S5. Source tracking results at layer five and layer six for microbiome samples from a less studied biome.** Left: source tracking results at the fifth layer; Right: source tracking results at the sixth layer. Results have shown that ONN4MST could identify the actual source from those polluted microbial communities. A_1, A_2: two samples collected from a single well; B_1, B_2: two samples collected from another single well; C_1, C_2: two samples collected from the third single well; D-H: samples collected from other five wells, respectively.


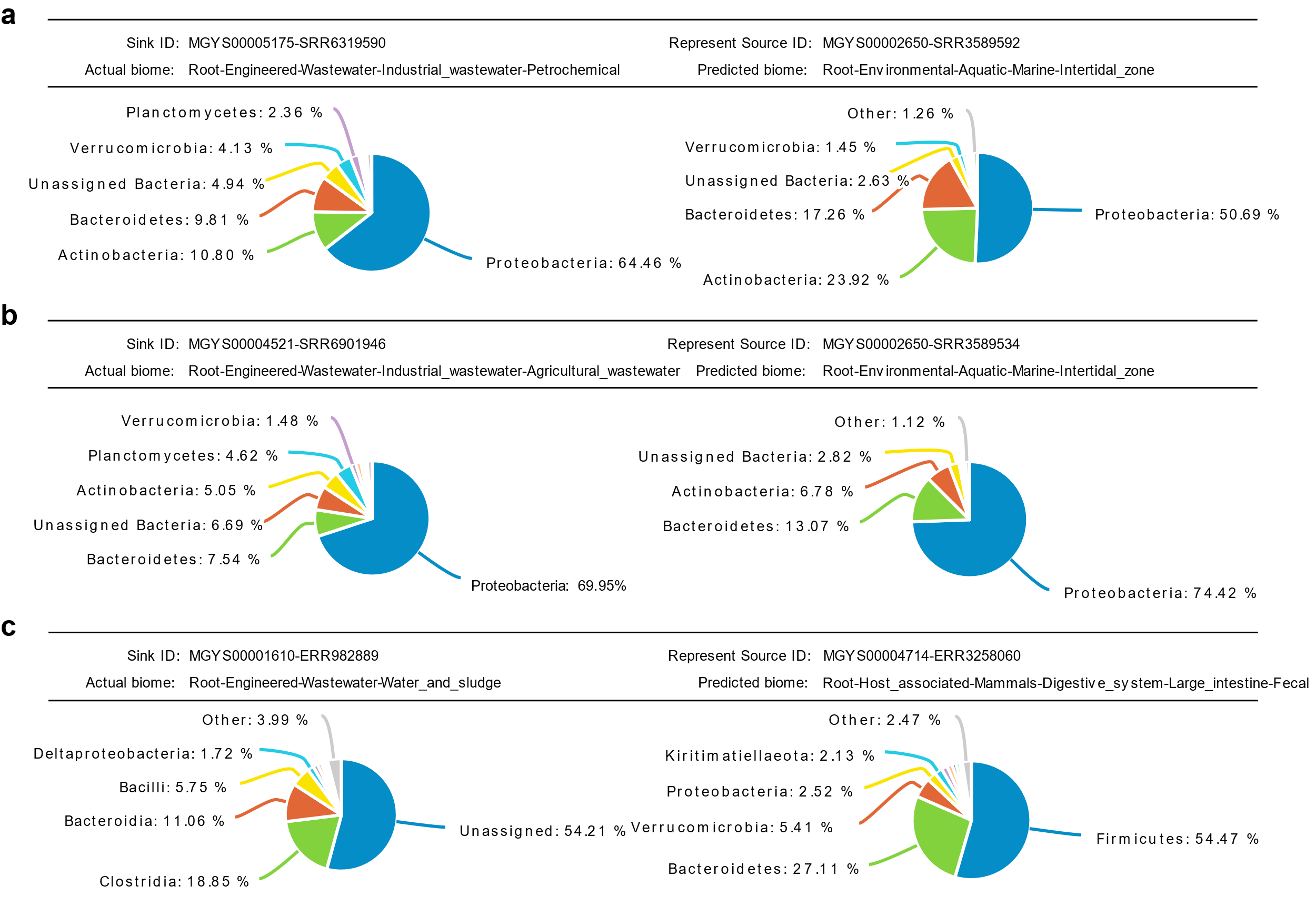


**Figure S6. Knowledge discovery of similar samples from ontologically-remote biomes. a.** Sample “MGYS00005175-SRR6319590” (from MGnify database) from biome “Root-Engineered-Wastewater-Industrial_wastewater-Petrochemical” has been identified by ONN4MST as similar with those from biome “Root-Environmental-Aquatic-Marine-Intertidal_zone”; **b.** Sample “MGYS000045215-SRR6901946” (from MGnify database) from biome “Root-Engineered-Wastewater-Industrial_wastewater-Agricultural_wastewater” has been identified by ONN4MST as similar with those from biome “Root-Environmental-Aquatic-Marine-Intertidal_zone”; **c.** Sample “MGYS00001610-ERR982889” (from MGnify database) with annotated biome “Root-Engineered-Wastewater-Water_and_sludge” have been identified by ONN4MST as similar with those from biome “Root-Host_associated-Mammals-Digestive_system-Large_intestine-Fecal”.

**Table S1. Microbial community samples and data used for model building and testing.** Notes: Human*: refers to those samples selected from MGnify biome “Host_associated-Human”. Aquatic**: refers to those samples selected from MGnify biome “Aquatic”. Soil***: refers to those samples selected from MGnify biome “Environmental-Terrestrial” and biome “Plants-Rhizosphere”. FEAST: refers to those samples selected from Shenhav et al.

| **Dataset** | **Combined** | **Human** | **Water** | **Soil** | **FEAST** |
| --- | --- | --- | --- | --- | --- |
| **Top-level biome** | Root | Human* | Aquatic** | Soil*** | Human gut |
| **Number of biomes involved** | 114 | 25 | 44 | 16 | 3 |
| **Number of samples** | 125,823 | 53,553 | 27,667 | 11,528 | 10,270 |
| **Number of phyla** | 225 | 204 | 222 | 201 | 133 |
| **Number of classes** | 660 | 557 | 653 | 539 | 277 |
| **Number of orders** | 2,018 | 1,412 | 19,85 | 1,394 | 552 |
| **Number of families** | 6,232 | 2,801 | 6,040 | 2,962 | 1,118 |
| **Number of genera** | 16,081 | 6,523 | 15,261 | 6,753 | 3,389 |
| **Number of species** | 45,477 | 16,135 | 36,406 | 12,769 | 5,762 |
| **Average number of species per sample** | 411.22 | 408.55 | 350.23 | 532.12 | 111.05 |
| **Notes** | Selected samples from MGnify | Selected samples from MGnify biome “Human” | Selected samples from MGnify biome “Aquatic” | Selected samples from MGnify biome “Environmental-Terrestrial” and biome “Plants-Rhizosphere” | Selected samples from FEAST dataset^1^ |

**Table S2. Biomes and number of samples used in EBI MGnify and this study.** All samples shown in this table belong to the Combined dataset, while certain sets of samples belong to the Human, Water and Soil datasets, as shown in the second column of the table.

| **MGnify biome** | **Belong to** | **ONN4MST biome** | **Number of samples** |
| --- | --- | --- | --- |
| Root |  | Root | 0 |
| Abyssal_plane | Water | Root-Environmental-Aquatic-Marine-Oceanic-Abyssal_plane | 792 |
| Activated_Sludge |  | Root-Engineered-Wastewater-Activated_Sludge | 233 |
| Agricultural | Soil | Root-Environmental-Terrestrial-Soil-Agricultural | 1,132 |
|  | Soil | Root-Environmental-Terrestrial-Soil-Loam-Agricultural | 48 |
| Agricultural_land | Soil | Root-Environmental-Terrestrial-Soil-Crop-Agricultural_land | 16 |
| Agricultural_wastewater |  | Root-Engineered-Wastewater-Industrial_wastewater-Agricultural_wastewater | 34 |
| Alkaline | Water | Root-Environmental-Aquatic-Non_marine_Saline_and_Alkaline-Alkaline | 0 |
| Aphotic_zone | Water | Root-Environmental-Aquatic-Marine-Oceanic-Aphotic_zone | 62 |
| Aquatic | Water | Root-Environmental-Aquatic | 0 |
| Benthic | Water | Root-Environmental-Aquatic-Marine-Oceanic-Benthic | 292 |
| Biofilm | Water | Root-Environmental-Aquatic-Freshwater-Groundwater-Biofilm | 11 |
| Black_smokers | Water | Root-Environmental-Aquatic-Marine-Hydrothermal_vents-Black_smokers | 2 |
| Blood | Human | Root-Host_associated-Human-Circulatory_system-Blood | 24 |
| Boreal_forest | Soil | Root-Environmental-Terrestrial-Soil-Boreal_forest | 3 |
| Buccal_mucosa |  | Root-Host_associated-Mammals-Digestive_system-Oral_cavity-Buccal_mucosa | 26 |
| Cecum |  | Root-Host_associated-Mammals-Digestive_system-Large_intestine-Cecum | 201 |
| Circulatory_system | Human | Root-Host_associated-Human-Circulatory_system | 2 |
| Coastal | Water | Root-Environmental-Aquatic-Marine-Coastal | 4,307 |
| Cold_seeps | Water | Root-Environmental-Aquatic-Marine-Cold_seeps | 555 |
| Contaminated | Soil | Root-Environmental-Terrestrial-Soil-Contaminated | 1 |
| Coral_reef | Water | Root-Environmental-Aquatic-Marine-Intertidal_zone-Coral_reef | 1,004 |
| Crop | Soil | Root-Environmental-Terrestrial-Soil-Crop | 1 |
| Desert | Soil | Root-Environmental-Terrestrial-Soil-Desert | 28 |
| Diffuse_flow | Water | Root-Environmental-Aquatic-Marine-Hydrothermal_vents-Diffuse_flow | 1 |
| Digestive_system |  | Root-Host_associated-Mammals-Digestive_system | 4,941 |
|  | Human | Root-Host_associated-Human-Digestive_system | 1,810 |
|  |  | Root-Host_associated-Insecta-Digestive_system | 165 |
| Dissolved_organics_(aerobic) |  | Root-Engineered-Wastewater-Nutrient_removal-Dissolved_organics_(aerobic) | 48 |
| Dissolved_organics_(anaerobic) |  | Root-Engineered-Wastewater-Nutrient_removal-Dissolved_organics_(anaerobic) | 8 |
| Endophytes |  | Root-Host_associated-Plants-Rhizoplane-Endophytes | 35 |
|  |  | Root-Host_associated-Plants-Phylloplane-Endophytes | 3,025 |
| Engineered |  | Root-Engineered | 0 |
| Environmental |  | Root-Environmental | 0 |
| Epiphytes |  | Root-Host_associated-Plants-Rhizosphere-Epiphytes | 36 |
| Estuary | Water | Root-Environmental-Aquatic-Marine-Intertidal_zone-Estuary | 83 |
| Fecal |  | Root-Host_associated-Mammals-Digestive_system-Fecal | 4,822 |
|  |  | Root-Host_associated-Mammals-Digestive_system-Large_intestine-Fecal | 2,904 |
|  | Human | Root-Host_associated-Human-Digestive_system-Large_intestine-Fecal | 29,427 |
| Female | Human | Root-Host_associated-Human-Reproductive_system-Female | 20 |
| Foregut |  | Root-Host_associated-Mammals-Digestive_system-Foregut | 0 |
| Forest_soil | Soil | Root-Environmental-Terrestrial-Soil-Forest_soil | 230 |
|  | Soil | Root-Host_associated-Plants-Rhizosphere-Forest_soil | 959 |
| Freshwater | Water | Root-Environmental-Aquatic-Freshwater | 9 |
| Grasslands | Soil | Root-Environmental-Terrestrial-Soil-Grasslands | 350 |
| Groundwater | Water | Root-Environmental-Aquatic-Freshwater-Groundwater | 0 |
| Host-associated |  | Root-Host_associated | 0 |
| Human | Human | Root-Host_associated-Human | 1,016 |
| Hydrothermal_vents | Water | Root-Environmental-Aquatic-Marine-Hydrothermal_vents | 52 |
| Hypersaline | Water | Root-Environmental-Aquatic-Non_marine_Saline_and_Alkaline-Hypersaline | 7 |
| Industrial_wastewater |  | Root-Engineered-Wastewater-Industrial_wastewater | 261 |
| Insecta |  | Root-Host_associated-Insecta | 608 |
| Intertidal_zone | Water | Root-Environmental-Aquatic-Marine-Intertidal_zone | 90 |
| Intestine | Human | Root-Host_associated-Human-Digestive_system-Intestine | 553 |
| Lake | Water | Root-Environmental-Aquatic-Freshwater-Lake | 3,475 |
| Large_intestine | Human | Root-Host_associated-Human-Digestive_system-Large_intestine | 535 |
|  |  | Root-Host_associated-Mammals-Digestive_system-Large_intestine | 494 |
| Loam | Soil | Root-Environmental-Terrestrial-Soil-Loam | 0 |
| Lympathic_system | Human | Root-Host_associated-Human-Lympathic_system | 0 |
| Lymph_nodes | Human | Root-Host_associated-Human-Lympathic_system-Lymph_nodes | 12 |
| Mammals |  | Root-Host_associated-Mammals | 0 |
| Mangrove_swamp | Water | Root-Environmental-Aquatic-Marine-Intertidal_zone-Mangrove_swamp | 106 |
| Marginal_Sea | Water | Root-Environmental-Aquatic-Marine-Marginal_Sea | 220 |
| Marine | Water | Root-Environmental-Aquatic-Marine | 5,922 |
| Microbial_mats | Water | Root-Environmental-Aquatic-Marine-Hydrothermal_vents-Microbial_mats | 11 |
| Microbialites | Water | Root-Environmental-Aquatic-Marine-Intertidal_zone-Microbialites | 7 |
| Mine_water |  | Root-Engineered-Wastewater-Industrial_wastewater-Mine_water | 79 |
| Mixed |  | Root-Mixed | 7,231 |
| Nasal_cavity | Human | Root-Host_associated-Human-Respiratory_system-Nasopharyngeal-Nasal_cavity | 17 |
| Nasopharyngeal | Human | Root-Host_associated-Human-Respiratory_system-Nasopharyngeal | 2,637 |
| Neritic_zone | Water | Root-Environmental-Aquatic-Marine-Neritic_zone | 77 |
| Non-marine_Saline_and_Alkaline | Water | Root-Environmental-Aquatic-Non_marine_Saline_and_Alkaline | 0 |
| Nutrient_removal |  | Root-Engineered-Wastewater-Nutrient_removal | 0 |
| Oceanic | Water | Root-Environmental-Aquatic-Marine-Oceanic | 3,457 |
| Oil-contaminated | Water | Root-Environmental-Aquatic-Marine-Oceanic-Oil_contaminated | 69 |
| Oil-contaminated_sediment | Water | Root-Environmental-Aquatic-Marine-Oil_contaminated_sediment | 256 |
| Oil-contaminated_sediments | Water | Root-Environmental-Aquatic-Marine-Oceanic-Oil_contaminated_sediments | 48 |
| Oil_seeps | Water | Root-Environmental-Aquatic-Marine-Oil_seeps | 2 |
| Oral | Human | Root-Host_associated-Human-Digestive_system-Oral | 5,820 |
| Oral_cavity |  | Root-Host_associated-Mammals-Digestive_system-Oral_cavity | 0 |
| Pelagic | Water | Root-Environmental-Aquatic-Marine-Pelagic | 176 |
| Periodontal_pockets | Human | Root-Host_associated-Human-Digestive_system-Oral-Periodontal_pockets | 10 |
| Permafrost | Soil | Root-Environmental-Terrestrial-Soil-Permafrost | 74 |
| Petrochemical |  | Root-Engineered-Wastewater-Industrial_wastewater-Petrochemical | 14 |
| Pharynx | Human | Root-Host_associated-Human-Respiratory_system-Nasopharyngeal-Pharynx | 55 |
| Photic_zone | Water | Root-Environmental-Aquatic-Marine-Oceanic-Photic_zone | 376 |
| Phylloplane |  | Root-Host_associated-Plants-Phylloplane | 21 |
| Plants |  | Root-Host_associated-Plants | 1,986 |
| Pulmonary_system | Human | Root-Host_associated-Human-Respiratory_system-Pulmonary_system | 0 |
| Reproductive_system | Human | Root-Host_associated-Human-Reproductive_system | 0 |
| Respiratory_system | Human | Root-Host_associated-Human-Respiratory_system | 23 |
| Rhizoplane |  | Root-Host_associated-Plants-Rhizoplane | 0 |
| Rhizosphere |  | Root-Host_associated-Plants-Rhizosphere | 3,925 |
| Root |  | Root-Host_associated-Plants-Root | 703 |
| Rumen |  | Root-Host_associated-Mammals-Digestive_system-Foregut-Rumen | 42 |
|  |  | Root-Host_associated-Mammals-Digestive_system-Stomach-Rumen | 612 |
| Saliva | Human | Root-Host_associated-Human-Digestive_system-Oral-Saliva | 3,862 |
| Salt_crystallizer_pond | Water | Root-Environmental-Aquatic-Non_marine_Saline_and_Alkaline-Salt_crystallizer_pond | 108 |
| Salt_marsh | Water | Root-Environmental-Aquatic-Marine-Intertidal_zone-Salt_marsh | 757 |
| Sand | Soil | Root-Environmental-Terrestrial-Soil-Sand | 16 |
| Sediment | Water | Root-Environmental-Aquatic-Marine-Coastal-Sediment | 979 |
|  | Water | Root-Environmental-Aquatic-Sediment | 315 |
|  | Water | Root-Environmental-Aquatic-Marine-Sediment | 3,031 |
|  | Water | Root-Environmental-Aquatic-Marine-Cold_seeps-Sediment | 82 |
|  | Water | Root-Environmental-Aquatic-Non_marine_Saline_and_Alkaline-Alkaline-Sediment | 98 |
|  | Water | Root-Environmental-Aquatic-Marine-Wetlands-Sediment | 105 |
|  | Water | Root-Environmental-Aquatic-Marine-Oceanic-Sediment | 468 |
|  | Water | Root-Environmental-Aquatic-Thermal_springs-Sediment | 10 |
|  | Water | Root-Environmental-Aquatic-Non_marine_Saline_and_Alkaline-Hypersaline-Sediment | 19 |
|  | Water | Root-Environmental-Aquatic-Marine-Neritic_zone-Sediment | 5 |
|  | Water | Root-Environmental-Aquatic-Marine-Intertidal_zone-Sediment | 140 |
|  | Water | Root-Environmental-Aquatic-Marine-Hydrothermal_vents-Sediment | 74 |
| Skin | Human | Root-Host_associated-Human-Skin | 4,848 |
| Soil | Soil | Root-Environmental-Terrestrial-Soil | 5,038 |
|  | Soil | Root-Host_associated-Plants-Rhizosphere-Soil | 3,380 |
| Sputum | Human | Root-Host_associated-Human-Respiratory_system-Pulmonary_system-Sputum | 135 |
| Stomach |  | Root-Host_associated-Mammals-Digestive_system-Stomach | 3 |
| Subgingival_plaque | Human | Root-Host_associated-Human-Digestive_system-Oral-Subgingival_plaque | 794 |
| Supragingival_plaque | Human | Root-Host_associated-Human-Digestive_system-Oral-Supragingival_plaque | 513 |
| Terrestrial |  | Root-Environmental-Terrestrial | 0 |
| Thermal_springs | Water | Root-Environmental-Aquatic-Thermal_springs | 0 |
| Throat | Human | Root-Host_associated-Human-Digestive_system-Oral-Throat | 21 |
| Tropical_rainforest | Soil | Root-Environmental-Terrestrial-Soil-Tropical_rainforest | 191 |
| Uranium_contaminated | Soil | Root-Environmental-Terrestrial-Soil-Uranium_contaminated | 1 |
| Vagina | Human | Root-Host_associated-Human-Reproductive_system-Vagina | 164 |
| Volcanic | Water | Root-Environmental-Aquatic-Marine-Volcanic | 1 |
| Wastewater |  | Root-Engineered-Wastewater | 110 |
| Water_and_sludge |  | Root-Engineered-Wastewater-Water_and_sludge | 508 |
| Wetlands | Soil | Root-Environmental-Terrestrial-Soil-Wetlands | 60 |
|  | Water | Root-Environmental-Aquatic-Marine-Wetlands | 6 |
| buccal_mucosa | Human | Root-Host_associated-Human-Digestive_system-Oral-buccal_mucosa | 289 |
| posterior_fornix | Human | Root-Host_associated-Human-Reproductive_system-Vagina-posterior_fornix | 11 |
| tongue_dorsum | Human | Root-Host_associated-Human-Digestive_system-Oral-tongue_dorsum | 955 |

**Number of samples in different datasets**

| **Dataset** | **Number of samples** |
| --- | --- |
| Combined | 125,823 |
| Human | 53,553 |
| Water | 27,667 |
| Soil | 11,528 |

*Note*: All samples shown in this table belong to the Combined dataset, while certain sets of samples belong to the Human, Water and Soil datasets, as shown in the second column of the table.

**Table S3. Evaluation of ONN4MST using the general model built based on the Combined dataset.**

| **Datasets** | **All features** | | | | | **Selected features** | | | | |
| --- | --- | --- | --- | --- | --- | --- | --- | --- | --- | --- |
|  | **Precision** | **Recall** | **Accuracy** | $\boldsymbol{F}_{\boldsymbol{max}}$ | **AUC** | **Precision** | **Recall** | **Accuracy** | $\boldsymbol{F}_{\boldsymbol{max}}$ | **AUC** |
| Human dataset | 0.917 | 0.651 | 0.988 | 0.804 | 0.978 | 0.947 | 0.853 | 0.994 | 0.914 | 0.993 |
| Soil dataset | 0.97 | 0.692 | 0.985 | 0.849 | 0.986 | 0.959 | 0.802 | 0.988 | 0.876 | 0.986 |
| Water dataset | 0.926 | 0.747 | 0.993 | 0.849 | 0.984 | 0.887 | 0.781 | 0.993 | 0.833 | 0.99 |
| FEAST dataset | 0.051 | 0.978 | 0.293 | 0.188 | 0.684 | 0.052 | 0.995 | 0.286 | 0.128 | 0.723 |

**Table S4. Evaluation of ONN4MST using the model trained on the human dataset.**

| **Datasets** | **All features** | | | | | **Selected features** | | | | |
| --- | --- | --- | --- | --- | --- | --- | --- | --- | --- | --- |
|  | **Precision** | **Recall** | **Accuracy** | $\boldsymbol{F}_{\boldsymbol{max}}$ | **AUC** | **Precision** | **Recall** | **Accuracy** | $\boldsymbol{F}_{\boldsymbol{max}}$ | **AUC** |
| Soil dataset | 1 | 0 | 0.895 | 0.19 | 0.5 | 1 | 0 | 0.895 | 0.19 | 0.5 |
| Water dataset | 1 | 0 | 0.976 | 0.047 | 0.5 | 1 | 0 | 0.976 | 0.047 | 0.5 |

**Table S5. Evaluation of simple neural network on all five datasets.** Note: For each dataset, we used the model trained on that dataset for evaluation. The evaluation procedure of the ONN model is illustrated in Supplementary Fig. 1c and described in Methods. ONN4MST based on all features and selected features were both evaluated at the bottom (sixth) layer with a threshold of 0.5. (Abbreviations. Pr: Precision, Rc: Recall, Acc: Accuracy).

| **Dataset** | **All features** | | | | | **Selected features** | | | | |
| --- | --- | --- | --- | --- | --- | --- | --- | --- | --- | --- |
|  | Pr | Rc | Acc | *Fmax* | AUC | Pr | Rc | Acc | *Fmax* | AUC |
| All | 0.932 | 0.083 | 0.996 | 0.328 | 0.695 | 0.913 | 0.092 | 0.997 | 0.386 | 0.716 |
| Human | 0.953 | 0.154 | 0.988 | 0.447 | 0.679 | 0.933 | 0.255 | 0.991 | 0.461 | 0.745 |
| Soil | 0.5 | 0 | 0.997 | 0.103 | 0.708 | 1 | 0 | 0.997 | 0.581 | 0.731 |
| Water | 0.877 | 0.122 | 0.993 | 0.379 | 0.721 | 0.901 | 0.113 | 0.994 | 0.399 | 0.791 |
| FEAST | 0.818 | 0.293 | 0.970 | 0.592 | 0.890 | 0.833 | 0.375 | 0.972 | 0.649 | 0.953 |

**Table S6.** **Results of five biome from “Human” using all features by ONN4MST at fifth layer.** The results have shown that ONN4MST could still reach a high AUC when the number of samples decrease from five thousand to five hundred.

| **Biome** | **Number of samples** | **Precision** | **Recall** | **Accuracy** | **AUC** |
| --- | --- | --- | --- | --- | --- |
| “Root-Host_associated-Human-Digestive_system-Oral” | 5820 | 0.932 | 0.965 | 0.990 | 0.996 |
| “Root-Host_associated-Human-Respiratory_system-Nasopharyngeal” | 2637 | 0.951 | 0.897 | 0.997 | 0.992 |
| “Root-Host_associated-Human-Digestive_system-Large_intestine” | 535 | 0.936 | 0.955 | 0.974 | 0.994 |
| “Root-Host_associated-Human-Reproductive_system-Vagina” | 164 | 0.714 | 0.754 | 0.999 | 0.962 |

**Table S7. Running time (s) of all methods when performing source tracking with one query against different datasets.** Note: For the Striped UniFrac, assuming there are N queries and M source samples, we estimate the time/memory cost to be N*M, rather than (N+M)^2^.

| **Datasets to be searched** | **ONN4MST** | **ONN4MST_FS** | **FEAST** | **SourceTracker** | **JSD** | **Meta-Prism** | **Striped UniFrac** |
| --- | --- | --- | --- | --- | --- | --- | --- |
| FEAST | 0.13 | 0.034 | 21,876 | 821,066 | 12.93 | 0.03 | 1.62 |
| Soil | 0.14 | 0.037 | 32,077 | 736,789 | 14.72 | 0.04 | 1.81 |
| Water | 0.17 | 0.034 | 113,330 | 1,548,727 | 20.88 | 0.19 | 4.36 |
| Human | 0.19 | 0.033 | 274,066 | 3,692,956 | 49.25 | 0.39 | 8.46 |
| Combined | 0.18 | 0.036 | 481,442 | 7,960,753 | 137.05 | 0.93 | 19.72 |
| 1M | 0.18 | 0.036 | 3,826,343 | 63,269,457 | 1,095 | 7.97 | 156.71 |

**Table S8. Running time (s) of all methods when performing source tracking with different sizes of testing sets on Combined dataset.** Note: For the Striped UniFrac, assuming there are N queries and M source samples, we estimate the time/memory cost to be N*M, rather than (N+M)^2^.

| **Number of queries** | **ONN4MST** | **ONN4MST_FS** | **FEAST** | **SourceTracker** | **JSD** | **Meta-Prism** | **Striped UniFrac** |
| --- | --- | --- | --- | --- | --- | --- | --- |
| 1 | 0.18 | 0.04 | 481,442 | 7,960,753 | 142.50 | 0.94 | 19.70 |
| 100 | 18.85 | 2.28 | 48,146,202 | 796,080,733 | 14,250 | 93.90 | 1,969.90 |
| 10,000 | 1,726.41 | 120.01 | 4,814,630,598 | 79,608,074,239 | 1,425,000 | 9,388 | 196,985 |
| 1,000,000 | 224,993.90 | 10,177.20 | 481,463,060,786 | 7,960,807,427,834 | 142,500,000 | 938,830 | 19,698,460 |

**Table S9.** **Memory utilization (GB) of all methods when performing source tracking with one query against different datasets.** Note: For the Striped UniFrac, assuming there are N queries and M source samples, we estimate the time/memory cost to be N*M, rather than (N+M)^2^.

| **Datasets to be searched** | **ONN4MST** | **ONN4MST_FS** | **FEAST** | **SourceTracker** | **JSD** | **Meta-Prism** | **Striped UniFrac** |
| --- | --- | --- | --- | --- | --- | --- | --- |
| FEAST | 7.58 | 1.72 | 3.02 | 1.14 | 1.97 | 0.13 | 0.01 |
| Soil | 8.14 | 1.73 | 7.20 | 2.17 | 5.91 | 0.14 | 0.01 |
| Water | 7.85 | 1.74 | 18.00 | 4.61 | 11.83 | 0.28 | 0.02 |
| Human | 7.58 | 1.77 | 36.00 | 9.78 | 23.65 | 0.52 | 0.05 |
| Combined | 22.24 | 1.85 | 84.00 | 18.28 | 47.30 | 1.15 | 0.11 |
| 1M | 22.24 | 1.85 | 671.61 | 141.12 | 364.35 | 9.06 | 0.86 |

**Table S10. Memory utilization (GB) of all methods when performing source tracking with different sizes of testing sets on Combined dataset.** Note: For the Striped UniFrac, assuming there are N queries and M source samples, we estimate the time/memory cost to be N*M, rather than (N+M)^2^.

| **Number of queries** | **ONN4MST** | **ONN4MST_FS** | **FEAST** | **SourceTracker** | **JSD** | **Meta-Prism** | **Striped UniFrac** |
| --- | --- | --- | --- | --- | --- | --- | --- |
| 1 | 22.24 | 1.85 | 84.00 | 18.28 | 47.30 | 1.17 | 0.11 |
| 100 | 22.50 | 1.85 | 84.00 | 18.28 | 47.30 | 1.27 | 0.20 |
| 10,000 | 27.32 | 2.07 | 84.00 | 18.28 | 47.30 | 1.46 | 9.48 |
| 1,000,000 | 504.27 | 23.44 | 84.00 | 375.92 | 47.30 | 1.60 | 938.58 |

**Table S11. The prediction results 303 samples from diverse human body sites.** These 303 samples include 90 gut samples, 183 oral samples and 30 vaginal samples.

| **Biome ontology** | | **Layer 2** | **Layer 3** | **Layer 4** | **Layer 5** |
| --- | --- | --- | --- | --- | --- |
| Gut | Biome | Host_associated | Human | Digestive_system | Large_intesetine |
|  | Accuracy | 100% | 100% | 100% | 100% |
| Oral | Biome | Host_associated | Human | Digestive_system | Oral |
|  | Accuracy | 100% | 100% | 84.2% | 44.3% |
| Vaginal | Biome | Host_associated | Human | Reproductive_system | Vagina |
|  | Accuracy | 100% | 66.7% | 0% | 0% |

**Table S12. Average source contributions from Mammals (pets) and Soil for indoor house environments, quantified by self-defined ONN4MST.** For kitchen environments, the proportions of inputs from Mammal and Soil are around 1.0% and 1.1%.

|  | Bathroom_  Door_Knob | Dog_Nose | Front_  Door_Knob | Kitchen_  Counter | Kitchen_  Floor | Kitchen_  Light_Switch |
| --- | --- | --- | --- | --- | --- | --- |
| Mammal | 0.7% | 1.3% | 0.0% | 1.7% | 1.2% | 0.1% |
| Soil | 0.1% | 0.0% | 0.0% | 0.1% | 0.2% | 3.1% |

**Table S13. The prediction results of 148 samples from ceca of bird by using ONN4MST.** “Bird” represents a newly introduced biome which belongs to the biome of “Host_associated”, and these 148 samples from ceca of bird are not in the Combined dataset.

| **Biome ontology** | **Layer 2** | **Layer 3** | | **Layer 4** |
| --- | --- | --- | --- | --- |
| Predicted biome | Host_associated | Human | Mammals | Digestive_system |
| No. samples | 148 | 115 | 33 | 148 |

**Table S14. The prediction results for 203 gut microbiome samples of the Hadza hunter-gatherers of Tanzania by using ONN4MST.** These 203 gut microbiome samples are divided into “Dry” and “Wet” categories, in which 106 samples are from “Dry” category meaning samples are collected from human in dry seasons, and the other 97 samples are from “Wet” category meaning samples are collected from human in wet seasons**.**

| **Biome ontology** | **Layer 2** | | **Layer 3** | |
| --- | --- | --- | --- | --- |
| Predicted biome | Host_associated | Environmental | Human | Aquatic |
| No. samples in “Dry” category as queries | 95 | 11 | 95 | 11 |
| No. samples in “Wet” category as queries | 67 | 30 | 67 | 30 |

**Table S15. The open searching results by using ONN4MST against the Combined dataset.** Several remotely similar samples among biomes “Engineered”, “Host_associated” and “Environmental” were discovered.

| **Sample ID in Mgnify** | **Actual biome** | **Predicted biome by using ONN4MST** | **Contribution** |
| --- | --- | --- | --- |
| MGYS00005175-SRR6314145 | Root-Engineered-Wastewater-Industrial_wastewater-Petrochemical | Root-Environmental-Aquatic-Marine-Coastal-Sediment | 0.9045 |
| MGYS00005175-SRR6319590 | Root-Engineered-Wastewater-Industrial_wastewater-Petrochemical | Root-Environmental-Aquatic-Marine-Intertidal_zone | 0.9916 |
| MGYS00001653-ERR1201972 | Root-Engineered-Wastewater-Water_and_sludge | Root-Host_associated-Mammals-Digestive_system-Large_intestine-Fecal | 0.9914 |
| MGYS00001652-ERR1201420 | Root-Engineered-Wastewater-Water_and_sludge | Root-Host_associated-Human-Digestive_system-Large_intestine-Fecal | 0.9976 |
| MGYS00001610-ERR982892 | Root-Engineered-Wastewater-Water_and_sludge | Root-Host_associated-Mammals-Digestive_system-Large_intestine-Fecal | 0.955 |
| MGYS00001652-ERR1201415 | Root-Engineered-Wastewater-Water_and_sludge | Root-Host_associated-Human-Digestive_system-Large_intestine-Fecal | 0.9316 |
| MGYS00001653-ERR1201940 | Root-Engineered-Wastewater-Water_and_sludge | Root-Host_associated-Mammals-Digestive_system-Large_intestine-Fecal | 0.9684 |
| MGYS00001652-ERR1201412 | Root-Engineered-Wastewater-Water_and_sludge | Root-Host_associated-Human-Digestive_system-Large_intestine-Fecal | 0.9957 |
| MGYS00001610-ERR982938 | Root-Engineered-Wastewater-Water_and_sludge | Root-Host_associated-Mammals-Digestive_system-Large_intestine-Fecal | 0.9914 |
| MGYS00001653-ERR1201941 | Root-Engineered-Wastewater-Water_and_sludge | Root-Host_associated-Mammals-Digestive_system-Large_intestine-Fecal | 0.955 |
| MGYS00001653-ERR1201974 | Root-Engineered-Wastewater-Water_and_sludge | Root-Host_associated-Mammals-Digestive_system-Large_intestine-Fecal | 0.9143 |
| MGYS00001653-ERR1201957 | Root-Engineered-Wastewater-Water_and_sludge | Root-Host_associated-Mammals-Digestive_system-Large_intestine-Fecal | 0.9233 |
| MGYS00001610-ERR982888 | Root-Engineered-Wastewater-Water_and_sludge | Root-Host_associated-Mammals-Digestive_system-Large_intestine-Fecal | 0.988 |
| MGYS00001610-ERR982933 | Root-Engineered-Wastewater-Water_and_sludge | Root-Host_associated-Mammals-Digestive_system-Large_intestine-Fecal | 0.972 |
| MGYS00001610-ERR982934 | Root-Engineered-Wastewater-Water_and_sludge | Root-Host_associated-Mammals-Digestive_system-Large_intestine-Fecal | 0.9683 |
| MGYS00001652-ERR1201417 | Root-Engineered-Wastewater-Water_and_sludge | Root-Host_associated-Human-Digestive_system-Large_intestine-Fecal | 0.9982 |
| MGYS00001653-ERR1201939 | Root-Engineered-Wastewater-Water_and_sludge | Root-Host_associated-Mammals-Digestive_system-Large_intestine-Fecal | 0.9792 |
| MGYS00001652-ERR1201410 | Root-Engineered-Wastewater-Water_and_sludge | Root-Host_associated-Human-Digestive_system-Large_intestine-Fecal | 0.9849 |
| MGYS00001610-ERR982890 | Root-Engineered-Wastewater-Water_and_sludge | Root-Host_associated-Mammals-Digestive_system-Large_intestine-Fecal | 0.9792 |
| MGYS00001610-ERR982917 | Root-Engineered-Wastewater-Water_and_sludge | Root-Host_associated-Mammals-Digestive_system-Large_intestine-Fecal | 0.9233 |
| MGYS00001610-ERR982932 | Root-Engineered-Wastewater-Water_and_sludge | Root-Host_associated-Mammals-Digestive_system-Large_intestine-Fecal | 0.9721 |
| MGYS00001610-ERR982940 | Root-Engineered-Wastewater-Water_and_sludge | Root-Host_associated-Mammals-Digestive_system-Large_intestine-Fecal | 0.9143 |
| MGYS00001610-ERR982887 | Root-Engineered-Wastewater-Water_and_sludge | Root-Host_associated-Mammals-Digestive_system-Large_intestine-Fecal | 0.9944 |
| MGYS00001610-ERR982889 | Root-Engineered-Wastewater-Water_and_sludge | Root-Host_associated-Mammals-Digestive_system-Large_intestine-Fecal | 0.9976 |
| MGYS00001610-ERR982898 | Root-Engineered-Wastewater-Water_and_sludge | Root-Host_associated-Mammals-Digestive_system-Large_intestine-Fecal | 0.913 |
| MGYS00001610-ERR982891 | Root-Engineered-Wastewater-Water_and_sludge | Root-Host_associated-Mammals-Digestive_system-Large_intestine-Fecal | 0.9684 |
| MGYS00001610-ERR982896 | Root-Engineered-Wastewater-Water_and_sludge | Root-Host_associated-Mammals-Digestive_system-Large_intestine-Fecal | 0.9474 |
| MGYS00001586-ERR701617 | Root-Engineered-Wastewater-Industrial_wastewater | Root-Host_associated-Human-Digestive_system-Large_intestine-Fecal | 0.9034 |
| MGYS00001555-ERR380862 | Root-Engineered-Wastewater-Industrial_wastewater | Root-Host_associated-Mammals-Digestive_system-Stomach-Rumen | 0.9334 |

**Table S16. Databases and software parameters used in this study.**

| **Database** |  |  |
| --- | --- | --- |
| **Database** | **Author** | **Website** |
| NCBI taxdump (released Feb 1, 2019) | [Federhen S, 2012](https://www.ncbi.nlm.nih.gov/pmc/articles/PMC3245000/) | https://ftp.ncbi.nlm.nih.gov/pub/taxonomy/taxdump_archive/ |
| **Software** | | |
| **Software** | **Author** | **Website** |
| textmineR (version 3.0.4) | [Tommy Jones](https://www.rtextminer.com/) | https://github.com/TommyJones/textmineR |
| Striped-Unifrac (committed Aug 1, 2018) | [McDonald et al., 2018](https://www.nature.com/articles/s41592-018-0187-8) | https://github.com/biocore/unifrac |
| Meta-Prism (version 2.0) | [Mo Zhu, Kai Kang and Kang Ning, 2020](https://academic.oup.com/bib/advance-article-abstract/doi/10.1093/bib/bbaa009/5729211) | https://github.com/HUST-NingKang-Lab/Meta-Prism-2.0 |
| SourceTracker (version 1.0) | [Knights, D. Kuczynski, J. et al., 2011](https://www.nature.com/articles/nmeth.1650) | https://github.com/danknights/sourcetracker |
| FEAST (committed Aug 30, 2019) | [Shenhav, L., Thompson, M. et al., 2019](https://www.nature.com/articles/s41592-019-0431-x) | https://github.com/cozygene/FEAST |
| Python (version 3.7.4) | [Oliphant and Travis E, 2007](https://ieeexplore.ieee.org/abstract/document/4160250/) | https://www.python.org |
| Pandas (version 1.0.1) | [McKinney, W. et al., 2010](https://www.semanticscholar.org/paper/Data-Structures-for-Statistical-Computing-in-Python-McKinney/f6dac1c52d3b07c993fe52513b8964f86e8fe381) | https://pandas.pydata.org |
| Treelib (version 1.5.5) | [Xiaming Chen](https://github.com/caesar0301) | https://github.com/caesar0301/treelib |
| Numpy (version 1.16.1) | [Stéfan van der Walt et al., 2011](https://aip.scitation.org/doi/abs/10.1109/mcse.2011.37) | https://numpy.org |
| Scikit-learn (version 0.23.2) | [Pedregosa et al., 2011](http://www.jmlr.org/papers/volume12/pedregosa11a/pedregosa11a.pdf) | https://scikit-learn.org |
| Tensorflow (version 1.14.0) | [Martín Abadi et al., 2015](https://arxiv.org/abs/1603.04467) | https://www.tensorflow.org |
| R (version 3.6.1) | [Team, R. C., 2013](http://invalid.uri/) | https://www.r-project.org |
| **Parameters** |  |  |
| **Software** | **Parameter** | **Value** |
| RandomForestRegressor | n_estimators | 100 |
|  | random_state | 1 |
|  | max_depth | 10 |
| ONN4MST | activation function | ReLU |
|  | Optimizer | Adam |
|  | loss function | Sigmoid_cross_entropy_with_logits |
|  | training batch size | 512 |
|  | learning rate | 1.00E-04 |
|  | iterations | 30,000 |
| FEAST | different_source_flag | 1 |
|  | EM_iterations | 1,000 |
| SourceTracker | alpha1 | 0.001 |
|  | alpha2 | 0.001 |
| JSD | COUVERAGE | 1,000 |
| Meta-Prism | matrix | |
|  | cores | 5 |
| Striped UniFrac | m | Weighted_normalized |
|  | n | 30 |
