# Supplementary material for: Ontology-aware deep learning enables ultrafast and interpretable source tracking among sub-million microbial community samples from hundreds of niches
Source: Genome Med. 2022 Apr 26;14:43. doi: 10.1186/s13073-022-01047-5 (PMC9040266; doi:10.1186/s13073-022-01047-5)
Supplement: Supplementary file 3 — Additional file 3. Supplementary method about the data representation and other source tracking methods used in this study. [file 13073_2022_1047_MOESM3_ESM.docx]

**Additional File 3**

**Supplementary Method: Data representation**

Before generating fixed the Matrix for each microbial community sample, we constructed a phylogenetic tree using all taxa involved in the Combined dataset. The phylogenetic tree was used to calculate the relative abundance of taxa in each microbial community sample. A detailed procedure was shown below.

1. Construct phylogenetic tree using all taxa involved in the Combined dataset. Level 2 to level 9 of the phylogenetic tree represent eight taxonomical levels (“sk”, “k”, “p”, “c”, “o”, “f”, “g”, and species “s”), respectively.
2. Map abundance data of taxa to the phylogenetic tree.
3. Update abundance for each taxon in the phylogenetic tree, the abundances of descendants were also added. E.g. the abundance of “sk__Bacteria;k__;p__Actinob
   acteria” was also added to “sk__Bacteria”.
4. Calculate relative abundance for each taxon in the phylogenetic tree.
5. Fill the relative abundance of taxa at seven taxon levels (“sk”, “k”, “p”, “c”, “o”, “f”, and “g”) into the Matrix.
6. Finally, the format of the Matrix is $\left[ \begin{aligned} R_{t_{sk}^{g_{1}}}, R_{t_{k}^{g_{1}}},R_{t_{p}^{g_{1}}},R_{t_{c}^{g_{1}}},R_{t_{o}^{g_{1}}},R_{t_{f}^{g_{1}}},R_{t_{g}^{g_{1}}} \\ R_{t_{sk}^{g_{2}}}, R_{t_{k}^{g_{2}}},R_{t_{p}^{g_{2}}},R_{t_{c}^{g_{2}}},R_{t_{o}^{g_{2}}},R_{t_{f}^{g_{2}}},R_{t_{g}^{g_{2}}} \\ \vdots\vdots\vdots\vdots\vdots\vdots\vdots\\ R_{t_{sk}^{g_{n}}}, R_{t_{k}^{g_{n}}},R_{t_{p}^{g_{n}}},R_{t_{c}^{g_{n}}},R_{t_{o}^{g_{n}}},R_{t_{f}^{g_{n}}},R_{t_{g}^{g_{n}}} \end{aligned} \right]$.

Where $t_{sk}^{g_{n}}$ is a taxon in lineage of $g_{n}$ in the taxonomical level “sk”, $R_{t}$ is relative abundance of taxon $t$.

For instance, for a sample consists of three OTUs with the number of reads 1,639, 240 and 121 assigned respectively to three taxa “sk_Bacteria”, “sk_Bacteria;k_;p_Bacteroidetes”, and “sk_Bacteria;k_;p_Chlamydiae”, their abundances were firstly updated to 2,000 (1,639+240+121), 240, and 121, then normalized to form the Matrix $\left[ \begin{matrix} 1 & 1 & 0.12 & 0 & 0 & 0 & 0 \\ 1 & 1 & 0.06 & 1 & 1 & 1 & 1 \\ \vdots& \vdots& \vdots& \vdots& \vdots& \vdots& \vdots\end{matrix} \right]$, in which 0.06= 121/2,000, and 0.12= 240/2,000.

**Supplementary Method: Other source tracking methods used in this study**

Jensen Shannon Divergence (JSD) is a distance-based approach for comparison of two probability distributions. Firstly, JSD distance was calculated between each pair of microbial community samples. After JSD distances are computed for all pairs of microbial community samples, the normalized average similarity scores (computed using one minus JSD distance) of each microbial community sample to every biome source are regarded as the source contributions.

Striped UniFrac (Unique Fraction metric) is a phylogenetic-based distance measurement for microbial community samples. It calculates the distance as the fraction of the branch length of the tree that leads to descendants from either one environment or the other, but not both. Striped UniFrac can also be used to produce a distance matrix for the pairwise phylogenetic distances between the sets of samples.

FEAST is a microbial source tracking method based on the Expected Maximization (EM) algorithm. It processes microbial community samples as follows: the features (abundance of taxa) are firstly normalized, then the FEAST program with default parameters is conducted. Finally, FEAST program outputs source contributions.

SourceTracker is a microbial source tracking method based on the Bayesian algorithm. It processes microbial community samples as follows: the features (abundance of taxa) are firstly normalized, then the depth of the rarefaction is set to 1,000 when conducting the program. Finally, SourceTracker program outputs source contributions.

Meta-Prism is microbial community samples searching system, using phylogenetic-based scoring algorithm to calculate similarity score between two microbial community samples. Firstly, for each pair of microbial samples, Meta-Prism sums up their common component’s proportion based on the phylogenetic relationship of species recursively from each leaf node to the “Root”. After similarity scores are computed for all pairs of samples, normalized similarity score of each sample to each biome source was regarded as source contributions.
